# Machine learning predictors of post‐radiotherapy cardiotoxicity in a Latin American breast cancer cohort: Clinical risk factors outperform cardiac dosimetry

**DOI:** 10.1002/acm2.70862

**Published:** 2026-10-14

**Authors:** Víctor Aguirre‐Bravo, Rodrigo El Far, María Herrera Ruiz, Sophia Gálvez Santelices, Pablo Munoz‐Schuffenegger, Tomás Merino, Sergio Diez Domingo, Marcelo E. Andia

**Affiliations:** ^1^ Radiation Safety UC CHRISTUS Santiago Chile; ^2^ Institute of Physics Pontificia Universidad Católica de Chile Santiago Chile; ^3^ Radiotherapy UC CHRISTUS Santiago Chile; ^4^ Department of Hematology‐Oncology School of Medicine Pontificia Universidad Católica de Chile Santiago Chile; ^5^ Department of Physiology Universitat de València Valencia Spain; ^6^ Department of Radiology School of Medicine Pontificia Universidad Católica de Chile Santiago Chile; ^7^ Millennium Institute for Intelligent Healthcare Engineering (iHEALTH) Santiago Chile

**Keywords:** AHA PREVENT score, breast cancer radiotherapy, cardiac dosimetry, cardio‐oncology, Firth logistic regression, Latin America, machine learning, post‐radiotherapy cardiotoxicity

## Abstract

**Background:**

Most evidence on post‐radiotherapy cardiotoxicity (CDT) derives from Global North cohorts, and the relative contribution of baseline clinical risk versus cardiac dosimetry remains poorly characterized in Latin American populations.

**Purpose:**

To examine, in a Chilean breast cancer cohort, whether baseline clinical risk factors or cardiac dose‐volume metrics better predict post‐RT cardiac events, using supervised and unsupervised learning.

**Methods:**

Retrospective cohort of 42 breast cancer patients treated with thoracic radiotherapy (RT, median heart dose 2.6 Gy; median follow‐up 84.4 months). CDT was defined per CTCAE v5.0 (≥ Grade 1), excluding Grade 1 events coded under the non‐specific term *“Cardiac disorders – Other, specify”*, which on review were incidental imaging findings. Dose‐volume histogram (DVH) parameters from seven cardiac substructures were extracted. Firth penalized logistic regression was used for prediction, principal component analysis (PCA) and kernel PCA for exploratory clustering, and bootstrap 0.632+ for optimism correction. The 2023 American Heart Association (AHA) PREVENT score was evaluated post hoc.

**Results:**

Ten patients (23.8%) developed CDT and 16 (38.1%) experienced cardiovascular events. No DVH differences were found between CDT+ and CDT− groups across any cardiac structure (all p>0.05), although the design is powered only for large effects. By contrast, IMRT delivered significantly higher cardiac doses than 3D‐CRT across multiple substructures, without a differential CDT rate. Pre‐existing cardiovascular disease was the strongest predictor (crude OR = 6.50, p=0.020; adjusted OR = 2.08, 95% CI 1.03–4.16, p=0.040). The median time to the first CTCAE‐graded event was 48.4 months. The clinical‐only model reached an area under the curve (AUC) of 0.783 versus 0.604 for dosimetry alone; the combined model corrected to 0.649 (95% CI 0.204–0.933). The AHA PREVENT score showed no significant risk gradient (trend p=0.203) but was markedly less susceptible to overfitting than DVH‐only models (optimism 0.089 vs. 0.194).

**Conclusions:**

In this low‐cardiac‐dose cohort, baseline clinical variables outperformed cardiac dose‐volume metrics in predicting post‐RT cardiac events, although the design was not powered to exclude a dose‐effect relationship at these exposures. Registry‐based baseline risk assessment is therefore the more robust starting point in this setting, and IMRT breast RT planning warrants multi‐substructure cardiac dosimetry monitoring.

## 1 INTRODUCTION

In Chile, as across Latin America, cancer is projected to displace cardiovascular disease as the leading cause of mortality within the next decade.^1^, ^2^ Radiotherapy (RT) remains a cornerstone in the management of thoracic malignancies, but its success in improving long‐term survival has brought post‐RT cardiotoxicity (CDT) to the forefront of survivorship care.^3^, ^4^

The development of CDT encompasses a complex pathophysiological spectrum—from early subclinical myocardial dysfunction to clinical conditions such as coronary artery disease, valvular dysfunction, and heart failure—that can manifest years or even decades after treatment.^5^, ^6^, ^7^, ^8^ The predictive potential of cardiac dosimetry was foundationally established by Darby et al.,^9^ who demonstrated a linear relationship between the mean heart dose and major coronary events, with a 7.4% increase in risk per Gy. Subsequent research, such as that by van den Bogaard et al.,^10^ validated this risk but emphasized the superior predictive power of specific substructures, particularly the left ventricle (LV) $V_{5}$ metric. Recently, the field has evolved toward more sophisticated paradigms; studies by Talebi et al.^11^ and Dincer et al.^12^ demonstrate that machine learning models integrating high‐dimensional data—such as radiomics and dosiomics—significantly outperform traditional dosimetric metrics, reaching AUCs above 0.90 even in pilot‐scale cohorts.

Supervised and unsupervised learning offer a framework for synthesizing volumetric dosimetry with baseline clinical risk profiles, such as the AHA PREVENT score,^13^ to identify patients at risk. This is particularly relevant in Latin America, where health systems face the double burden of rising cancer incidence and a high prevalence of cardiovascular comorbidities.^14^

Furthermore, there is a persistent gap in the global scientific evidence. Characterizing geographic representation using the Global South/North framework^15^ — which distinguishes regions by their historical position in global scientific production rather than income thresholds alone — Global South populations remain significantly underrepresented in cardio‐oncology research.^16^ Most existing risk models are developed in Global North contexts, raising fundamental questions about their generalizability to populations with different epidemiological, genetic, and socio‐economic realities. Regional efforts to characterize radiotherapy‐induced cardiac toxicity through multimodal assessment—speckle‐tracking echocardiography, cardiac magnetic resonance and circulating biomarkers—have been initiated in this setting,^17^ but published cohorts pairing multi‐substructure cardiac dosimetry with clinical outcomes remain absent from the region. Generating local evidence is therefore essential to inform regional clinical guidelines and ensure that precision medicine in oncology benefits all survivors, regardless of geographic location.

This pilot study asks whether baseline clinical risk or cardiac substructure dosimetry better predicts post‐RT cardiac events in a Chilean breast cancer cohort, using supervised and unsupervised learning. Its aim is not a validated model but the identification of the most informative variables and a replicable protocol for larger‐scale cardio‐oncology research in the region.

## 2 MATERIALS AND METHODS

### 2.1 Study design and patient selection

This study was designed as an exploratory retrospective cohort analysis with a preliminary predictive focus, aimed at identifying clinical and dosimetric variables associated with post‐RT CDT. The study was conducted at the Radiotherapy Service of UC CHRISTUS, a tertiary academic medical center in Santiago, Chile. Ethical approval and informed consent procedures are detailed in the Ethics Statement section.

The study population was identified through convenience sampling of breast cancer patients who received RT between January 2017 and December 2023. Inclusion criteria were: (1) age ≥ 18 years; (2) histologically confirmed breast cancer; (3) completion of a thoracic RT course; (4) a minimum clinical follow‐up of 6 months; and (5) complete availability of both serum cardiac biomarkers and volumetric dosimetric data (including dose‐volume histograms and cardiac substructure segmentations).

Patients were excluded if follow‐up data were insufficient to determine the primary outcome or if any component of criterion (5) was unavailable; missing values in continuous clinical variables were imputed with the closest available value to the start of RT rather than excluded. The centre plans approximately 230 breast cancer patients for radiotherapy each year (191–304 annually; 908 patients over 2017–2020). Chart abstraction for this study was manual and was limited by the person‐hours available to an unfunded project: in practice it covered the patients simulated in January and February of each year, giving a screening frame of 169 patients, 88.6% of all breast cancer patients simulated in those two months. This operational ceiling, rather than any clinical criterion, is the principal determinant of cohort size. The two‐month window was not chosen for any clinical reason and is unrelated to cardiac risk; treating‐physician mix did not differ significantly between these months and the remainder of the year (p=0.065), and monthly breast volume was comparable (83.5 vs. 72.9 patients/month). Of the 169 patients screened, 42 formed the analysed cohort and 127 were excluded—overwhelmingly for reasons of outcome ascertainment rather than dosimetry. Fifty‐one (40.2%) had no documented post‐RT cardiac or clinical follow‐up, and 48 (37.8%) could not have their clinical record retrieved: the centre's transition from paper to electronic records is recent, and retrieval of archived paper charts carries a fee that this unfunded study could not meet. A further 12 (9.4%) were not treated for breast cancer at the centre, 7 (5.5%) had a plan that could not be extracted from the TPS, 4 (3.1%) had no recorded cardiac history and 5 (3.9%) were administrative errors. Failure to retrieve dose‐volume data therefore accounted for 5.5% of exclusions, against 78.0% attributable to outcome ascertainment; cardiac biomarkers, in turn, were never routine in the absence of a protocolized surveillance programme (Discussion) and were obtained on clinical indication or incidentally. Although the eligibility window extended through December 2023, the most recent RT completion date was August 2021. Substructure segmentations were additionally reviewed manually for a dataset frozen for this purpose. These sampling effects, rather than the single‐center design alone, drive both the size and the temporal composition of the cohort, and are the reason it is described as a convenience sample.

Laterality was not an exclusion criterion: right‐sided patients receive near‐zero cardiac dose and therefore constitute the internal low‐dose comparison group against which the dose–effect hypothesis is tested. Excluding them would have removed that contrast and reduced the cohort to 25 patients.

### 2.2 Treatment planning and delivery

RT was delivered on a single Varian Clinac 2100C/D linear accelerator (Varian Medical Systems, Palo Alto, CA, USA) using megavoltage photon beams (6 and 18 MV). This was the only accelerator in clinical use for breast radiotherapy at the centre during the study period, and it was verified as the treating unit in all 41 archived treatment plans retrieved for this study. Treatment simulation was performed using non‐contrast planning computed tomography (CT) with patients in the supine position, immobilized with dedicated breast boards.

Treatment plans were generated using the Eclipse treatment planning system version 8.6 (Varian Medical Systems, Palo Alto, CA, USA), used for all patients in this cohort. Delivery techniques were three‐dimensional conformal radiation therapy (3D‐CRT, n=33) and intensity‐modulated radiation therapy (IMRT, n=9). Technique and beam configuration were obtained from the archived DICOM treatment plans, available for 41 of the 42 patients; for the remaining patient both were taken from the clinical record. 3D‐CRT plans used opposed tangential fields (median 3 treatment fields, range 2–6), with enhanced dynamic wedges in 24 of 32 plans (wedge angles 10–45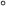) and field‐in‐field or mixed‐energy (6/18 MV) subfields at the same gantry angle in 23 of 32; an anterior supraclavicular field was planned separately in 7 patients and a sequential boost was delivered in 18. IMRT plans were fixed‐gantry sliding‐window multi‐field plans (median 6 fields, range 4–12) delivered at 6 MV without wedges. The larger number of incident directions in the IMRT plans distributes a low‐dose bath over a wider thoracic volume, which is the mechanism underlying the technique comparison reported in Section 3.2. Most patients received moderately hypofractionated schedules, with standard, hyperfractionated and ultra‐hypofractionated schemes in selected cases (Table S12). Plans were optimized so that planning target volume (PTV) coverage complied with institutional protocols, under cardiac constraints of mean heart dose ≤ 5 Gy (target < 3 Gy) and ipsilateral lung $V_{20}<$ 20%. A boost to the tumor bed was delivered when clinically indicated.

Regional nodal irradiation was not recorded categorically and was reconstructed from the planned dose to the ipsilateral level III axillary structure. The distribution was strongly bimodal (26 patients ≤ 7.3 Gy, 14 between 35.6 and 53.7 Gy), allowing unambiguous classification at a 20 Gy threshold: 14 patients received nodal irradiation and 26 breast or chest‐wall irradiation alone; in two the nodal structures were unavailable.

### 2.3 Clinical and dosimetric parameters

Clinical data, including demographics, cardiovascular risk factors (arterial hypertension, diabetes mellitus, smoking, sedentary lifestyle, alcohol consumption, body mass index [BMI] and obesity status, total and LDL cholesterol [hypercholesterolemia], and family history of cardiovascular disease), pre‐existing cardiac disease or cardiovascular events, and prior oncological history (previous cancer diagnosis, previous chemotherapy, and previous radiotherapy), were extracted from electronic health records.

For the dosimetric analysis, treatment plans were retrieved from local servers to export dose‐volume histograms (DVHs). Seven cardiac substructures (whole heart, left and right ventricles, left and right atria, left anterior descending [LAD] artery and thoracic aorta) were automatically contoured using AutoContour (version 2.6.5; Radformation, New York, NY, USA), then manually reviewed and edited against the Radiation Therapy Oncology Group (RTOG) consensus atlas for breast RT,^18^ which also served as the reference for a visual quality‐control review of structure labeling and dose‐volume consistency in all 42 patients. Mean dose ($D_{mean}$), maximum dose ($D_{max}$) and fractional volumes from $V_{5}$ to $V_{70}$ were extracted for each structure (Tables S2–S4).

### 2.4 Outcome definitions: CDT and cardiovascular events

Two independent outcomes were predefined:

1. Post‐RT CDT (Primary): Defined as any cardiac event documented in the electronic health record post‐RT, graded according to the Common Terminology Criteria for Adverse Events (CTCAE) version 5.0,^19^ *excluding* events coded under the non‐specific term *“Cardiac disorders – Other, specify”*. Cases were considered positive if they presented with at least one Grade ≥1 event meeting that definition (n=10, 23.8%).
2. Cardiovascular Events (Secondary): A broader definition encompassing (a) serum cardiac biomarkers (high‐sensitivity cardiac troponin T [hscTnT], high‐sensitivity cardiac troponin I [hscTnI], B‐type natriuretic peptide [BNP], and N‐terminal pro‐BNP [NT‐proBNP]) exceeding laboratory reference ranges post‐RT; (b) imaging‐based findings of cardiac dysfunction (via echocardiography or cardiac magnetic resonance (CMR));^20^ or (c) any clinical documentation of a cardiovascular event during follow‐up.

The non‐specific term was excluded because, on case‐by‐case review, the 11 events so coded (in 7 patients) were incidental imaging findings rather than diagnosed cardiac disease, 6 of them arising from imaging requested for an unrelated indication (Table S1). Since the cohort was not under systematic cardiology surveillance, their ascertainment was opportunistic. For 2 of the 7 patients this was the only recorded event, so they are CDT− under the primary definition.

Two further definitions were retained as pre‐specified sensitivity analyses: (${\mathrm{CDT}}_{A}$) any Grade ≥1 event, including the non‐specific term (n=12, 28.6%), the definition used in the originally submitted analysis; and (${\mathrm{CDT}}_{B}$) Grade ≥2 events only (n=4, 9.5%). Results are reported in Section 4 and Table S8.

### 2.5 Selection of predictor variables

Predictor variables were pre‐specified prior to analysis to restrict the hypothesis space and prevent overfitting, given the limited sample size (n = 42). Variables were organized into two independent sets according to their information domain, following the empirical Events Per Variable (EPV ≥ 5–10) rule:^21^

- Set 1 — Clinical: Four variables selected for their biological plausibility and availability in the cohort: age at the time of RT (continuous), pre‐existing cardiac disease or cardiovascular events (dichotomous), prior chemotherapy (CTx, dichotomous), and hypertension (HTN, dichotomous).
- Set 2 — Dosimetric: Three dosimetric parameters derived from the DVH and supported by evidence from systematic reviews: mean heart dose (Heart $D_{mean}$, Gy), the percentage of heart volume receiving ≥ 5 Gy (Heart $V_{5}$, %), and the mean dose to the left anterior descending artery (LAD $D_{mean}$, Gy). Correlations among the dose‐volume metrics are shown in Figure S3.

Seven variables across two sets gave a minimum EPV of 2.5–4.0 for the outcome with the fewest events (CDT, n=10), compatible with Firth penalized regression. RT technique was considered but excluded because of its collinearity with Heart $V_{5}$ (Spearman ρ=+0.38, p=0.013); CDT rates were technique‐independent (p=1.000; Section 3.2). For the combined model, two variables were retained from each set, chosen by the deviation of the odds ratio from unity in the domain‐specific multivariable models rather than by the raw odds ratio: Heart $D_{mean}$ and Heart $V_{5}$ for both outcomes. A sensitivity analysis substituting LAD $D_{mean}$ is reported in Table S13.

As a post‐hoc complementary analysis, the AHA PREVENT 2023 score (Predicting Risk of cardiovascular disease EVENTs)^22^ was evaluated as a categorical classifier of baseline cardiovascular risk from standard registry variables (age, sex, systolic blood pressure, lipids, estimated glomerular filtration rate and diabetes mellitus).

### 2.6 Statistical analyses

Continuous variables were expressed as medians and interquartile ranges (IQR), and categorical variables as frequencies and percentages; univariate comparisons used Fisher's exact test and the Mann‐Whitney U test. Event‐free survival was estimated by the Kaplan‐Meier method, with time to event measured from completion of RT to the event or study cutoff (March 1, 2026). Because the cohort combined hypofractionated, standard and hyperfractionated schedules, prescribed dose and Heart $D_{mean}$ were also converted to the equivalent dose in 2‐Gy fractions, EQD2=D×(d+α/β)/(2+α/β), with α/β=3 Gy for late‐responding cardiac tissue;^9^ for the heart, d is Heart $D_{mean}$ divided by the number of delivered fractions rather than the dose per fraction prescribed to the target, so that its EQD2 falls below its physical dose. This served as a sensitivity check across fractionation schemes (Section 3.1).

Natural separability was explored by Principal Component Analysis (PCA)^23^ and Kernel PCA^24^ with a radial basis function kernel, followed by K‐means clustering; agreement between clusters and the observed outcome was quantified by the Adjusted Rand Index (ARI).^25^ A binary partition (k=2) was used throughout to match the two‐class outcome, a choice supported by silhouette analysis^26^ across k=2–5 (Table S7).

Multivariate analysis used Firth penalized logistic regression,^27^, ^28^ which penalizes the likelihood by the square root of the Fisher information and thereby remains stable at low EPV and under separation (L‐BFGS‐B optimization; *SciPy* v1.13). Three nested models were fitted per outcome—Clinical (Set 1), Dosimetric (Set 2) and Combined—and the incremental value of dosimetry over clinical information was tested by Likelihood Ratio Test (LRT). Internal validation used bootstrap 0.632+^29^ (1,000 iterations) for optimism‐corrected AUC; calibration was assessed by the Brier score^30^ and the Hosmer‐Lemeshow test,^31^ and clinical utility by Decision Curve Analysis (DCA).^32^ The learning component of this study therefore comprises unsupervised structure discovery (PCA, kernel PCA and K‐means) and penalized supervised prediction with bootstrap internal validation; no radiomic or dosiomic image features were derived, and automated contouring is a data‐preparation step rather than part of the predictive analysis. Processing was performed in Python (v3.12) and R (v4.3.0) with the *logistf* package. During the preparation of this manuscript, the authors used Claude (Anthropic) to improve the language and readability of the text; all content was reviewed and edited by the authors, who take full responsibility for the publication.

#### 2.6.1 Precision of the design

No a priori sample size calculation was performed: this is an exploratory pilot study whose size was determined by data availability. To characterize what the design can resolve, we ran a precision analysis by simulation (4000 replicates) using the same two‐sided Fisher exact test applied throughout, holding the observed incidence fixed. With 42 patients and 10 events, the study has 80% power only for large associations (odds ratio ≥ 9.5 at the observed 28.6% exposure prevalence); the odds ratios of 2–3 commonly reported in cardio‐oncology are not detectable. Positive findings are therefore interpretable, whereas null findings—in particular the absence of dose‐volume differences between outcome groups—are inconclusive rather than evidence of absence. By the criteria of Riley et al.,^33^ a four‐parameter model at this prevalence would require approximately 87–340 patients depending on the anticipated Cox‐Snell $R^{2}$; the present cohort is well below that threshold, which is why the models are reported as hypothesis‐generating rather than as a validated prediction tool.

## 3 RESULTS

### 3.1 Patient characteristics and cardiovascular events

The cohort comprised 42 women (median age 59 years) followed for a median of 84.4 months [IQR: 72.4–96.6]. During this period, 10 patients (23.8%) developed CDT (primary outcome) and 16 (38.1%) experienced at least one cardiovascular event (CVD Events, secondary outcome). Event‐free survival for both outcomes is shown in Figure 2.

**FIGURE 1:**
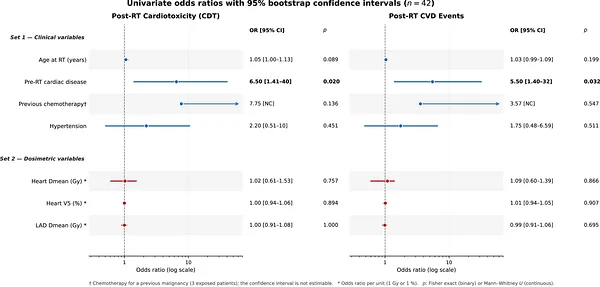
Effect magnitude (univariate odds ratio with 95% bootstrap CI) of clinical and dosimetric variables for the two pre‐specified outcomes (post‐RT CDT and CVD Events; n=42). OR >1 indicates increased relative risk; detailed estimates are provided in the Supplementary Material.

**FIGURE 2:**
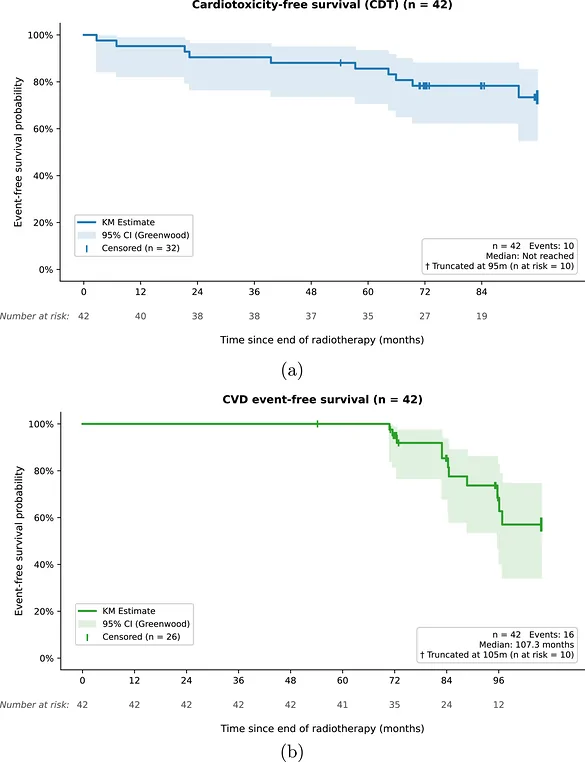
Kaplan–Meier event‐free survival for (a) post‐RT CDT and (b) post‐RT CVD Events. Shaded areas: 95% CI (Greenwood method); vertical marks: censored patients. Curves are truncated at the last time point with ≥10 patients at risk.

Baseline characteristics and univariate comparisons are summarized in Table 1, and the corresponding univariate effect magnitudes in Figure 1 (detailed estimates in Table S6). Patients in the CDT+ group were older and had a higher BMI, though neither difference reached significance, and they had a significantly higher prevalence of pre‐existing cardiovascular disease (60.0% vs. 18.8%; p=0.020). The prescribed RT dose was higher in the CDT+ group without reaching significance (p=0.055), and reflects a mixture of fractionation schemes rather than a biological dose difference: after EQD2 conversion (α/β=3 Gy; Section 2.6) neither the prescribed dose (p=0.452) nor Heart $D_{mean}$ (p=0.779) differed between groups, consistent with the physical‐dose comparison below.

**TABLE 1:** Baseline characteristics of the cohort (n=42), stratified by presence of post‐RT CDT.

Variable	No. CDT (n=32)	CDT (n=10)	p‐value
Demographics and anthropometrics			
Age (years)	53.0 [43.8–66.0]	63.5 [53.5–72.0]	0.089
BMI (kg/$m^{2}$)^a^	25.5 [23.3–27.5]	30.2 [26.0–31.7]	0.060
Cardiovascular risk factors			
Hypertension	10 (31.2%)	5 (50.0%)	0.451
Smoking (active/ex)	11 (34.4%)	4 (40.0%)	1.000
Diabetes mellitus	4 (12.5%)	2 (20.0%)	0.616
Pre‐RT cardiac disease	6 (18.8%)	6 (60.0%)	**0.020**
Treatment characteristics			
Left‐sided treatment	20 (62.5%)	5 (50.0%)	0.714
Chemotherapy (current episode)^c^	18 (56.2%)	2 (20.0%)	0.071
RT technique (3D‐CRT)^b^	25 (78.1%)	8 (80.0%)	1.000
RT dose (Gy)^d^	40.0 [40.0–42.5]	42.5 [40.6–42.5]	0.055
Follow‐up (months)	84.3 [72.2–95.9]	86.5 [82.9–104.7]	0.469
Dosimetry			
Heart volume (${\mathrm{cm}}^{3}$)^e^	513.7 [488.1–568.2]	593.6 [518.1–696.0]	0.111
Heart $D_{\text{mean}}$ (Gy)^e^	2.41 [1.72–4.17]	2.73 [2.58–3.08]	0.757
Heart $V_{5}$ (%)^e^	6.52 [0.73–13.54]	6.94 [6.15–10.51]	0.894
LV volume (${\mathrm{cm}}^{3}$)^e^	159.3 [144.4–172.9]	171.8 [163.6–214.2]	0.054
LV $D_{\text{mean}}$ (Gy)^e^	3.85 [0.91–5.08]	3.57 [2.19–4.40]	0.353
LAD $D_{\text{mean}}$ (Gy)^e^	10.31 [1.50–17.55]	6.65 [2.21–21.32]	1.000

*Note*: Categorical variables expressed as n (%); continuous variables as median [IQR]. p: Fisher's exact test for categorical variables; Mann‐Whitney U test for continuous variables.

Abbreviations: CDT, post‐RT CDT; BMI, body mass index; RT, radiotherapy; 3D‐CRT, three‐dimensional conformal radiotherapy; IMRT, intensity‐modulated radiation therapy; CVD, cardiovascular disease; LV, left ventricle; LAD, left anterior descending artery; $D_{mean}$, mean dose; $V_{x}$, percentage of volume receiving ≥x Gy; IQR, interquartile range.

^a^ Data available for n=37 (5 missing records).

^b^ Remaining patients received IMRT (n=9). Delivery technique was available for all 42 patients.

^c^ Chemotherapy for the index breast cancer, with a documented regimen (neoadjuvant, adjuvant or concomitant); 3 patients had no regimen recorded and are counted as unexposed. Chemotherapy given for a *previous* malignancy is a separate variable (Section 2.5) and is reported in Table S6.

^d^ RT Dose corresponds to the dose prescribed by the radiation oncologist to the primary target volume (whole breast or chest wall) prior to simulation, expressed in physical Gy. It does not include any separately delivered tumor bed boost (see Section 2.2 for boost indication); 24/41 patients with available data (58.5%) received a boost, recorded as a separate binary variable rather than an additional prescribed dose. Given the heterogeneous fractionation schemes in this cohort, the between‐group comparison of physical RT Dose should be interpreted alongside its EQD2‐converted counterpart (Section 3.1), which did not reach statistical significance (α/β=3 Gy).

^e^ DVH dose parameters extracted from the treatment planning system. Structural volumes (Heart, LV) from institutional treatment records. Heart and LV volumes were unavailable for one patient. Full DVH comparison across all cardiac substructures is provided in Table S2.

### 3.2 Characterization of post‐RT cardiovascular events and dosimetric context

Post‐RT cardiovascular events were characterized according to CTCAE v5.0 (Table S1 and Figure S1a; pre‐RT versus post‐RT terms in Figure S1c). Eleven distinct CTCAE terms were identified across the 10 patients with CDT, with a predominance of arrhythmic and conduction disorders (sinus bradycardia n=2 and sinus tachycardia n=1, all Grade 1; first‐degree and complete atrioventricular block, conduction disorder and atrial fibrillation, one patient each). Structural and other events were less common: left ventricular systolic dysfunction (n=1, Grade 3), aortic valve disease (n=2, Grade 1), mitral valve disease (n=1, Grade 2), heart failure (n=1, Grade 1) and myocardial infarction in two patients (Grade 2 and Grade 5, the latter fatal). A further 11 Grade 1 events in 7 patients were coded under the non‐specific term and are excluded from the primary outcome (Section 2.4), but retained in Table S1 for completeness. The median time from end of RT to the first CTCAE‐graded event was 48.4 months [IQR: 21.6–65.5], with only two of the ten CDT+ patients experiencing that first event within twelve months (Figure S1b, one row per patient).

No significant differences in DVH parameters were observed between CDT+ and CDT− groups across any of the seven cardiac substructures (Mann‐Whitney U, all p>0.05; Tables S2 and S4, Figure S2a,c), with median Heart $D_{mean}$ of 2.73 versus 2.41 Gy (p=0.757) and consistently low mean doses throughout. Analysis by delivery technique, in contrast, revealed significantly higher cardiac doses with IMRT (n=9) than with 3D‐CRT (n=33): Heart $V_{5}$ 26.56% versus 6.06% (p=0.001) and Heart $D_{mean}$ 4.26 versus 2.29 Gy (p=0.009). This low‐dose bath effect extended significantly to the right ventricle, left and right atria, and aorta (all p<0.05; Table S3 and Figure S2b). Despite these dosimetric differences, CDT rates were comparable between techniques (24.2% vs. 22.2%; Fisher exact p=1.000). These findings underscore the importance of multi‐substructure cardiac dosimetry monitoring in IMRT breast RT planning.

A sensitivity analysis restricted to the left‐sided subgroup (n=25: 5 CDT+, 20 CDT−; Table S5) found no DVH metric higher in CDT+ than in CDT−; LV $V_{5}$ and LV $D_{mean}$ were nominally lower in CDT+ (p=0.071 and p=0.169), opposite to the direction expected under a dose‐effect hypothesis.

### 3.3 Dimensionality analysis, clustering, and CDT prediction models

Unsupervised dimensionality reduction was used to explore whether patients separate naturally by outcome. Principal component analysis followed by K‐means clustering (k=2) yielded Adjusted Rand Index values near zero or slightly negative across all configurations (Table S9; highest ARI = 0.068, for the clinical set and CDT), indicating that the unsupervised partition does not recover the clinical groups with sufficient fidelity for diagnostic use. Explained variance by component, cluster‐validation indices and the K‐PCA projection are reported in Tables S7, S11 and Figure S4.

Kernel PCA with an RBF kernel, applied to capture non‐linear structure, produced no clear global separation either: it split the cohort into clusters of 18 and 24 patients, the lower‐risk one containing 1 of the 10 events (negative predictive value 94.4%, 95% exact binomial CI 72.7%–99.9%, against a cohort‐wide non‐event rate of 76.2%; specificity 53.1%). The gain over the baseline rate is modest and the partition is exploratory, but it locates what separation these data do contain in the baseline clinical profile—which motivated the post‐hoc evaluation of the AHA PREVENT score in Section 3.6.

### 3.4 Predictive modeling: Firth penalized logistic regression

Three Firth models were fitted per outcome to isolate the contribution of each information domain: *Model 1* (Clinical) and *Model 2* (Dosimetric) quantify what each achieves alone, and *Model 3* (Combined) tests via a nested Likelihood Ratio Test (LRT) whether integrating them adds discrimination beyond the clinical baseline; *Model 3* alone would leave that contrast unmeasured. Variables are those pre‐specified in Section 2.5, standardized as Z‐scores; reported ORs correspond to a one standard deviation increase or to binary presence, with Wald confidence intervals.

The contrast between domains is the central result. The *Clinical Model* alone achieved the highest discrimination for CDT, whereas the *Dosimetric Model* performed close to chance for both outcomes, with very wide confidence intervals reflecting the low EPV. Adding dosimetry to the clinical variables did not improve fit for either outcome (nested LRT p=0.43 and p=0.27). Pre‐existing cardiac disease was the strongest predictor throughout, and in *Model 3* for CDT it was the only variable whose confidence interval excluded the null. Comparative ROC curves are presented in Figure 3.

**FIGURE 3:**
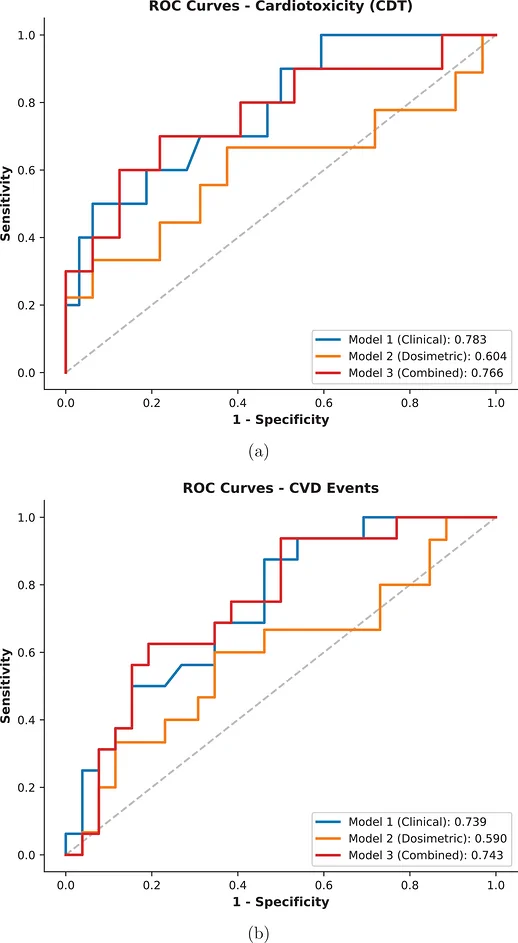
Comparative ROC curves for the three Firth models predicting (a) post‐RT CDT and (b) post‐RT CVD Events. Model 1: Clinical; Model 2: Dosimetric; Model 3: Combined.

### 3.5 Internal validation and clinical utility

Optimism correction of the *Combined Model* used the bootstrap 0.632+ estimator (B=1,000 resamples; Table 3). The apparent AUC fell to 0.649 (95% CI [0.204–0.933]) for CDT and 0.610 (95% CI [0.278–0.867]) for CVD Events; both corrected estimates exceed chance discrimination but their intervals are wide, as expected at n=42. Calibration, assessed primarily through the Brier score—which avoids arbitrary groupings and is more reliable at small sample sizes—showed a modest gain over the null model for both outcomes, with no evidence of lack of fit on the Hosmer‐Lemeshow test, although that test is underpowered here. Calibration plots across risk quintiles are provided in Figure S5.

**TABLE 2:** Firth penalized logistic regression results for both outcomes (n=42; 10 CDT events, 16 CVD events).

	Post‐RT CDT			Post‐RT CVD Events		
Variable	OR	95% CI	p	OR	95% CI	p
**Model 1 – Clinical** (AUC = 0.783 for CDT; 0.739 for CVD Events)						
Age at RT	1.51	0.60–3.85	0.383	1.32	0.60–2.93	0.490
Pre‐RT Cardiac Disease	1.85	0.91–3.79	0.092 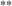	1.83	0.94–3.57	0.077 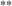
Previous CTx	1.34	0.67–2.69	0.407	1.11	0.56–2.20	0.765
Hypertension	1.05	0.44–2.51	0.918	1.01	0.47–2.18	0.981
**Model 2 – Dosimetric** ^a^ (AUC = 0.604 for CDT; 0.590 for CVD Events)						
Heart $D_{mean}$	0.74	0.04–15.73	0.849	0.36	0.02–5.90	0.472
LAD $D_{mean}$	1.23	0.44–3.42	0.689	0.93	0.37–2.37	0.883
Heart $V_{5}$	1.08	0.07–17.20	0.958	3.26	0.26–40.2	0.357
**Model 3 – Combined** (AUC = 0.766, LRT p=0.28 for CDT; 0.743, LRT p=0.27 for CVD Events)						
**Pre‐RT Cardiac Disease**	**2.08**	**1.03–4.16**	**0.040** 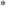	1.89	0.97–3.65	0.060^a^
Hypertension	1.39	0.65–2.96	0.393	—	—	—
Age at RT	—	—	—	1.32	0.65–2.69	0.438
Heart $D_{mean}$	3.18	0.30–33.92	0.338	0.80	0.09–7.31	0.845
Heart $V_{5}$	0.29	0.02–3.89	0.351	1.52	0.17–13.45	0.707

*Note*: OR values correspond to standardized variables (per 1 SD increase) or binary presence. LRT compares each combined model against its clinical variables alone. Dashes indicate variables not retained in that combined model. *Abbreviations*: OR, odds ratio; CI, Wald confidence interval; AUC, area under the curve; LRT, Likelihood Ratio Test; $D_{mean}$, mean dose; $V_{5}$, percentage of volume receiving ≥5 Gy.

^a^ *Model 2* was fitted on the 41 patients with complete dosimetric data: in one patient the LAD was not contoured on the treated plan. *Models 1* and *3* include all 42.

∗p<0.05; 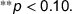

**TABLE 3:** Internal validation metrics (Model 3) using bootstrap 0.632+ and calibration analysis.

Outcome	Apparent AUC	Corrected AUC	Brier Score	Null Brier	H‐L p ^a^
CDT post‐RT	0.766	0.649	0.144	0.181	0.889
CVD Events	0.743	0.610	0.196	0.236	0.388

Abbreviations: AUC, area under the curve; Null Brier, Brier Score of intercept‐only model (reference baseline); H‐L p, Hosmer‐Lemeshow test p‐value.

^a^ H‐L test is reported as supplementary evidence; it has low statistical power at n=42 and should be interpreted with caution.

Decision Curve Analysis (DCA) for the Combined Model (Figure 4) showed positive net benefit over default strategies across the clinically relevant threshold range (∼1–65% for CDT and ∼15–45% for CVD Events), peaking near the event prevalence (∼25–30%). Notably, for CDT the clinical‐only model achieved net benefit comparable to the combined model, indicating that dosimetric parameters added little decision‐analytic value—consistent with the stronger predictive contribution of baseline clinical risk. As these curves reflect apparent (non–optimism‐corrected) net benefit at n=42, they are exploratory, supporting preliminary stratification rather than established clinical utility.

**FIGURE 4:**
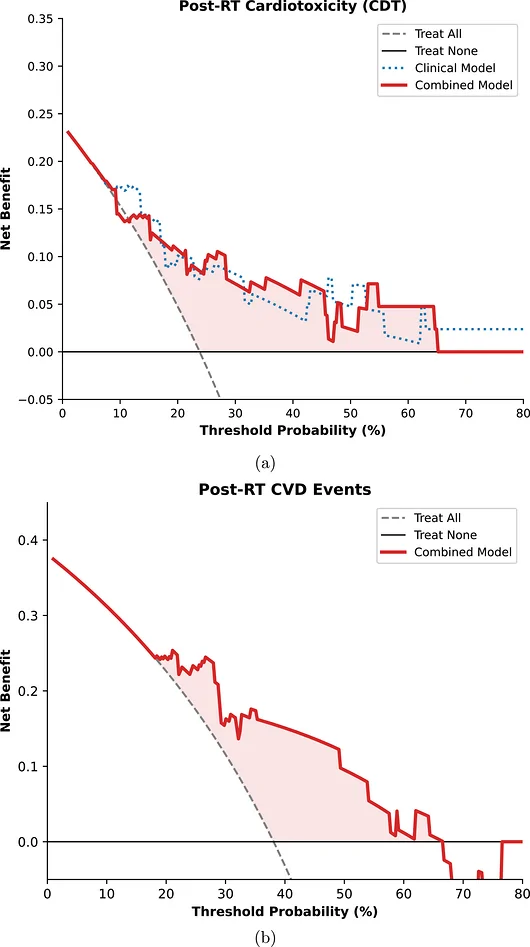
Decision curve analysis for the combined model predicting (a) post‐RT CDT and (b) post‐RT CVD events. Positive net benefit over default strategies across the clinically relevant range (∼1–65% CDT; ∼15–45% CVD events). Given n=42, curves are apparent (non–optimism‐corrected) and exploratory.

### 3.6 AHA PREVENT score analysis and predictive modeling

Motivated by the stronger contribution of baseline cardiovascular risk relative to dosimetry, and by the clinical‐profile structure suggested by K‐PCA (Section 3.3), the 2023 AHA PREVENT score^13^ was evaluated post hoc as a categorical classifier. The 42 cases fell into three collapsed categories—*Low* (<3%, n=17), *Borderline+Intermediate* (3–<10%, n=12) and *High* (≥10%, n=13); Figure 5a shows the continuous distribution with the applied cut‐offs. Observed CDT rates rose from the lowest category but without a monotonic gradient across the upper two: 11.8%, 33.3% and 30.8% (Figure 5b).

**FIGURE 5:**
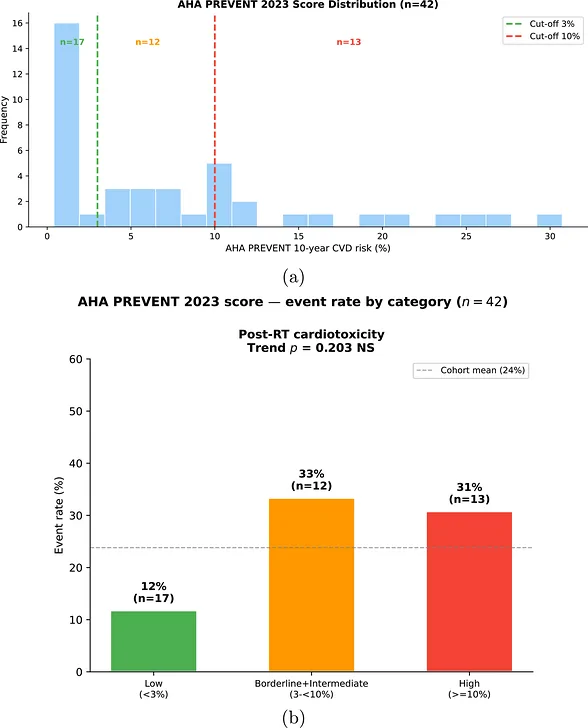
AHA PREVENT 2023 risk score analysis. (a) Continuous distribution across the cohort (n=42) with clinical cut‐offs. (b) Observed CDT rates by risk category; the increase over the *Low* category does not reach statistical significance (trend p=0.203).

The Cochran‐Armitage test did not detect a significant ordered trend for CDT (z=1.274; p=0.203, not significant [NS]; CDT‐free survival by category shown in Figure S6), nor for CVD Events (p=0.340, NS). In a categorical Firth regression with *Low* as reference (Table S10), both upper categories gave point estimates consistent with elevated CDT risk but intervals far too wide to establish it with 10 events; for CVD Events, the *Borderline+Intermediate* category showed the same direction without reaching significance (OR=4.09; p=0.082).

Apparent AUCs for CDT were similar across *Model A* (PREVENT categories, 0.641), *Model B* (DVH parameters, 0.569) and *Model C* (both, 0.706), with no significant pairwise difference (Figure 6). The informative comparison is not apparent discrimination but its stability. After bootstrap 0.632+ correction, *Model A* retained an AUC of 0.551 with an optimism of 0.089, whereas *Model B* fell to 0.375 (optimism 0.194) and *Model C* to 0.482 (optimism 0.225): the dosimetric models lose their entire apparent advantage under resampling and end below chance, while the registry‐based score, though only marginally better than chance, is the only one whose corrected estimate does not collapse. PREVENT is therefore best read here as an exploratory, more parsimonious alternative to dose‐volume metrics, not as a validated stratification tool.

**FIGURE 6:**
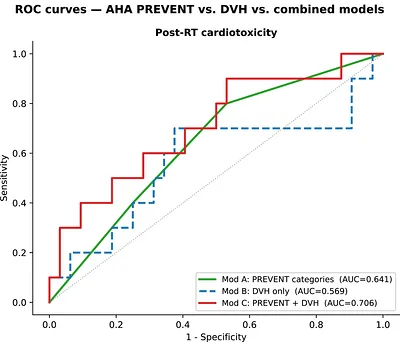
Comparative ROC curves for CDT models. ModA: PREVENT categories; ModB: DVH alone; ModC: PREVENT + DVH. Apparent discrimination is similar across models; Model A is distinguished by its lower optimism under bootstrap resampling (0.089 vs. 0.194 and 0.225).

## 4 DISCUSSION

The operational definition of the outcome deserves comment first, because it shapes everything that follows. CTCAE v5.0 Grade ≥1 is standardized and widely used, but it captures a broad spectrum ranging from subclinical findings to clinically significant events. In this cohort 11 of the 32 graded events were incidental imaging findings recorded under a non‐specific term (Section 2.4); including them inflates incidence in a way that depends on how often patients happen to be imaged rather than on cardiac risk. Restricting the primary outcome to specific CTCAE terms lowers the incidence from 28.6% to 23.8% and, as shown below, materially changes the temporal pattern. We retain the broader definition as a sensitivity analysis rather than discarding it, since the choice is a judgement about ascertainment and readers should be able to see both.

The predominance of arrhythmic and conduction disorders among the retained events provides mechanistic context: such events are primarily conditioned by pre‐existing inflammatory and fibrotic cardiovascular substrates.

The absence of significant DVH differences between CDT+ and CDT− patients should not, however, be read as evidence against a dose–effect relationship. In 910 patients with a median mean heart dose of 2.37 Gy—essentially the exposure recorded here—van den Bogaard et al.^10^ demonstrated a significant dose–effect for acute coronary events (16.5% per Gy). Our cohort is not powered to resolve effects of that magnitude (Section 2.6.1), so the null dosimetric finding reflects limited resolution rather than a protective sub‐threshold exposure regime.

This interpretation is supported externally. In 975 patients, Yang et al.^34^ found no difference in major cardiovascular events between right‐ and left‐sided irradiation, yet detected a laterality effect specifically among patients *without* baseline cardiovascular risk factors—indicating that the dosimetric signal is masked when baseline clinical burden is high, as in the present cohort. Consistently, IMRT delivered higher cardiac doses than 3D‐CRT across multiple substructures (Section 3.2) without a differential CDT rate, which nonetheless underscores the value of multi‐substructure dosimetric monitoring as follow‐up extends.

This direction deserves comment, since inverse planning is more commonly reported to *reduce* mean heart dose than tangential 3D‐CRT—though those comparisons pit competing plans against the same target volume, whereas ours is observational between patients whose targets differ. Here the effect is better explained by the structure of the planning constraints than by technique itself. Both techniques respected the 5 Gy hard tolerance (31/33 and 7/9 plans), but the 3 Gy objective was met by 25/33 (76%) of 3D‐CRT versus 2/9 (22%) of IMRT plans (12/19 versus 1/6 among left‐sided patients). With a hard limit at 5 Gy and only a soft objective at 3 Gy, inverse optimization has no incentive to push cardiac dose lower once nodal coverage and lung constraints compete, whereas tangential geometry achieves low cardiac dose incidentally; that IMRT met the ipsilateral lung $V_{20}$ constraint less often (median 21.3% versus 14.7%) suggests those cases were genuinely more demanding. Nodal irradiation is part of the explanation: IMRT was used preferentially for nodal cases (5/8 versus 9/32 among patients with both variables recorded, p=0.102), and the technique effect was confined to the nodal stratum (+1.99 Gy, p=0.042), being absent among patients treated to the breast or chest wall alone (+0.07 Gy, p=1.000, with only three IMRT patients). Nodal irradiation by itself did not raise mean heart dose (p=0.288), so the relevant factor is the combination of a more demanding target with an optimization that has no incentive to push cardiac dose below the 3 Gy objective.

Prescribed RT dose, the baseline variable closest to a difference between CDT groups, was assessed as a potential confounder of the DVH comparison: the difference disappeared after EQD2 conversion (Section 3.1), reflecting heterogeneous fractionation rather than a biological disparity. Neither prescribed dose nor cardiac volume correlated meaningfully with Heart $D_{mean}$ or $V_{5}$, consistent with cardiac dose constraints being optimized independently of cardiac anatomy and total prescribed dose. The absence of DVH differences between CDT groups therefore reflects genuine dosimetric equivalence rather than a confounding artifact.

The inverse direction of the left‐sided sensitivity analysis (Section 3.2), together with a higher upper‐IQR Heart $D_{mean}$ in the CDT− group (Table 1), is consistent with possible indication bias: cardiac dose may have been preferentially minimized in clinically vulnerable patients—sound practice that would also spuriously reinforce the appearance of no dosimetric effect.

The empirical results in this Chilean cohort confirmed that baseline clinical history carries a larger effect size than isolated dosimetric parameters. Pre‐existing cardiovascular disease was the most potent predictor and remained significant after multivariate adjustment (Figure 1, Table 2); its crude‐to‐adjusted attenuation is expected from simultaneous adjustment for age, hypertension and dosimetric covariates, reflecting confounding structure rather than a weakening signal.

The timing of events points the same way. Under the primary definition the median time to the first event was 48.4 months, with only two of ten patients affected in the first year—a late, cumulative pattern consistent with the natural history of radiation‐associated cardiac disease.^9^, ^10^ Under the broader definition the median was 21.8 months, and three of the four earliest events (6.2, 7.6 and 19.9 months) were precisely the non‐specific incidental findings; the earliest of all, at 2.7 months, is a Grade 1 valvular finding that qualifies and is retained. The apparently early onset was therefore largely an artefact of opportunistic detection rather than evidence of an early radiation effect; once removed, both the incidence and the latency of this cohort fall within the published range.

These results are best read against comparable model strata (Table 4). Cardiac exposure here matches van den Bogaard et al.^10^ and is about half that of the Darby et al.^9^ series. In Dincer et al.^12^—a cohort of identical size—and Talebi et al.,^11^ AUCs above 0.95 were reached only after adding radiomic or dosiomic image features, while their conventional clinical‐plus‐dosimetric models stayed near 0.67–0.85; our optimism‐corrected AUC of 0.649 sits at the lower end of that range. Wherever baseline cardiovascular status and cardiac dose have been compared directly—in Taiwan, Iran and Türkiye as well as here—clinical risk has carried the larger share of the predictive signal.

**TABLE 4:** Comparison with published cohorts relating cardiac radiation exposure to cardiac outcomes in breast cancer.

Study	Region	n	Outcome	Cardiac dose	Dominant predictor	Discrimination
Darby 2013^9^	Sweden/Denmark	2168	Major coronary events (case‐control)	MHD 4.9 Gy (mean)	Mean heart dose	+7.4% per Gy
van den Bogaard 2017^10^	Netherlands	910	Acute coronary events	MHD 2.37 Gy (median)	LV $V_{5}$, with age and clinical risk score	c = 0.83
Yang 2025^34^	Taiwan	975	Major CV events (10‐year)	not reported	Baseline CV risk (0.7% vs 13.7%); laterality NS	—
Talebi 2025^11^	Iran	83	Echocardiographic dysfunction at 6 months	not reported	Clinical variables over dosimetry	0.73 dosimetric; 0.85 + clinical; 0.97 + radiomics
Dincer 2025^12^	Türkiye	42	hs‐Troponin T elevation	not reported	Image‐derived features	0.67 clinical + dosimetric; 0.96 + radiomics
**Present study**	**Chile**	**42**	CTCAE v5.0 ≥ G1, specific terms (23.8%)	MHD 2.3–2.8 Gy (median)	Pre‐existing cardiovascular disease	0.783 clinical; 0.604 dosimetric; 0.649 corrected

*Abbreviations*: MHD, mean heart dose; LV, left ventricle; CV, cardiovascular; NS, not significant; c, concordance statistic.

To our knowledge this is the first Latin American cohort pairing multi‐substructure cardiac dosimetry with clinical outcomes; a search of the regional literature (LILACS/BVS) returned no comparable study.

That the unsupervised partitions do not recover the clinical groups (Section 3.3) indicates that the relationships between cardiovascular risk factors, dosimetric parameters and CDT in this cohort are of modest absolute magnitude. Consistently, the lower bound of the adjusted AUC for CDT (0.204) falls below 0.5, so the null hypothesis of non‐discrimination cannot be formally rejected with these data; the point estimates should be read as indicative signals rather than precision estimates.

Despite these uncertainties, the analysis consistently pointed to pre‐RT cardiovascular health rather than dose. The AHA PREVENT 2023 score^13^ did not achieve a statistically significant risk gradient here, and we therefore do not claim it as an actionable stratification tool. What it did show is a difference in stability: a third of the optimism of the DVH‐only model, and corrected discrimination that stayed above chance where dosimetry alone fell below it. A registry score built from data already in the clinical record is thus the more parsimonious and reproducible starting point in this setting—a hypothesis for larger cohorts to test, not a recommendation.

Pre‐existing cardiovascular disease remained the dominant predictor under both pre‐specified sensitivity definitions (Table S8). Notably, under the most restrictive definition (${\mathrm{CDT}}_{B}$, Grade ≥2 only, n=4) all four high‐grade events occurred exclusively in patients with pre‐existing cardiovascular disease (Fisher exact p=0.004): the clinical signal strengthens, rather than diminishes, with more restrictive criteria. A further post‐hoc analysis excluding the 13 events flagged in the record as related to a pre‐existing condition dissolves the association (p=0.387); we report it for transparency but not as evidence, since it is circular by construction—it preferentially removes events from precisely the patients whose baseline cardiovascular disease constitutes the exposure under study.

This study should be interpreted as exploratory, hypothesis‐generating work: its aim was not a validated model but the identification of high‐potential variables and a replicable protocol. Its limitations fall into four domains. *Statistical resolution*: the cohort is small (n=42; EPV 2.5–4.0, below the recommended 10–15) and powered only for large effects (Section 2.6.1), more than 30 tests were performed without correction for multiplicity, and no external validation was possible. *Design and ascertainment*: the single‐center, convenience‐sampled design (Section 2.1) restricts generalizability, and two resource constraints bound it further. Chart abstraction was limited by the person‐hours available to an unfunded study and covered only two months of each calendar year, so the screening frame is 169 of the 908 breast cancer patients planned over 2017–2020; year‐round abstraction would have enlarged the cohort several‐fold and is the single most consequential change for a successor study. Outcome ascertainment was constrained in turn by clinical records predating the centre's recent digitization, which are held in a physical archive whose retrieval carries a fee this study could not meet. Critically, patients were not enrolled in a protocolized cardio‐oncology surveillance programme: cardiac assessment followed clinical indication rather than fixed intervals, so ascertainment depended in part on how often each patient was imaged for other reasons. This motivated excluding non‐specific incidental findings from the primary outcome (Section 2.4), but residual events may still be under‐ascertained in patients followed less intensively, and subclinical dysfunction detectable only by systematic echocardiography or biomarkers would have been missed. *Unrecorded treatment detail*: cardioprotective medication was not captured; prior chemotherapy was recorded as binary, without distinguishing anthracyclines or anti‐HER2 agents; and cardiac‐sparing techniques (DIBH, prone positioning, gating) could not be retrieved, precluding attribution of the low cardiac doses observed. *Dosimetric uncertainty*: the dose‐calculation algorithm was not recorded per patient, although the planning system version restricts it to the Analytical Anisotropic Algorithm or pencil‐beam convolution; no geometric agreement metric between automated and edited contours was available, most relevant for the LAD; and the conduction system was not segmented, which may matter more for arrhythmic outcomes than whole‐chamber dose.^35^, ^36^ Future prospective studies should address these through standardized ascertainment of cardioprotective medication and cardiac‐sparing technique, structured cardiology surveillance aligned with current guidelines,^37^ dedicated conduction‐system delineation, and eligibility based on dose‐volume data and follow‐up alone, which would both enlarge the cohort and remove the dependence on opportunistic biomarker availability.

## 5 CONCLUSION

In this low‐cardiac‐dose Latin American cohort, baseline clinical vulnerability outperformed cardiac dosimetry in predicting post‐RT cardiac events—although the cohort was not powered to exclude a dose‐effect relationship at these exposures—while IMRT planning warrants multi‐substructure dosimetric monitoring. Registry‐based baseline risk assessment proved more stable under internal validation than dose‐volume metrics, but did not reach statistical significance and should be regarded as a hypothesis rather than a stratification tool. These hypothesis‐generating findings, drawn from an under‐represented Global South population,^15^ should be confirmed in larger prospective cohorts integrating biomarkers and standardized cardiology surveillance to advance prevention‐focused precision cardio‐oncology.

## AUTHOR CONTRIBUTIONS

This research was conceptualized by V.A.‐B. and M.E.A. Clinical expertise and cardio‐oncology guidance were provided by P.M.‐S. Medical physics expertise was contributed by R.E.F. Conceptual and methodological support was provided by S.D.D. Data collection and extraction were conducted by V.A.‐B., R.E.F., M.H.R., and S.G.S. Statistical analysis, predictive modeling, and interpretation of results were performed by V.A.‐B. and M.E.A. The manuscript was prepared by V.A.‐B. All authors contributed to the revision and validation of the final manuscript.

## FUNDING

This research received no specific grant for its conduct. The article processing charge was funded by the Millennium Institute for Intelligent Healthcare Engineering (iHEALTH), ANID — Millennium Science Initiative Program, ICN2021_004, and by FONDECYT Regular 1260578.

## CONFLICT OF INTEREST STATEMENT

The authors declare no conflicts of interest.

## ETHICS STATEMENT

This study was conducted in accordance with the ethical principles of the Declaration of Helsinki (1964) and its subsequent amendments. The research protocol was reviewed and approved by the Institutional Review Board (IRB) for Health Sciences at Pontificia Universidad Católica de Chile (approval ID: 250603001). Given the retrospective nature of the study and the use of de‐identified patient data, the IRB granted a waiver of individual informed consent. Patient confidentiality and data anonymization were maintained throughout all stages of analysis.

## Supporting information

Supporting file

## Data Availability

The data used and/or analyzed during the current study are available from the corresponding author on reasonable request.

## References

[1] World Cancer Research Fund . Global cancer incidence: both sexes ; 2026. Available from: https://www.wcrf.org/preventing‐cancer/cancer‐statistics/worldwide‐cancer‐data/#global‐cancer‐incidence‐both‐sexes

[2] Ministerio de Salud Gobierno de Chile . Plan Nacional de Cáncer 2018‐2028 . Ministerio de Salud Gobierno de Chile; 2018.

[3] Zamorano JL , Lancellotti P , Muñoz DR , et al. 2016 ESC position paper on cancer treatments and cardiovascular toxicity developed under the auspices of the ESC committee for practice guidelines. Eur Heart J. 2016;37(36):2768‐2801. doi:10.1093/eurheartj/ehw211 27567406

[4] Armenian SH , Lacchetti C , Barac A , et al. Prevention and monitoring of cardiac dysfunction in survivors of adult cancers: American society of clinical oncology clinical practice guideline. J Clin Oncol. 2017;35(8):893‐911. doi:10.1200/JCO.2016.70.5400 27918725

[5] Gagliardi G , Constine LS , Moiseenko V , et al. Radiation dose‐volume effects in the heart. Int J Radiat Oncol Biol Phys . 2010;76(3 SUPPL). doi:10.1016/j.ijrobp.2009.04.093 20171522

[6] Darby SC , Cutter DJ , Boerma M , et al. Radiation‐related heart disease: current knowledge and future prospects. Int J Radiat Oncol Biol Phys. 2010;76(3):656‐665. doi:10.1016/j.ijrobp.2009.09.064 PMC3910096 20159360

[7] Sardar P , Kundu A , Chatterjee S , et al. Long‐term cardiovascular mortality after radiotherapy for breast cancer: a systematic review and meta‐analysis. Clin Cardiol. 2017;40(2):73‐81. doi:10.1002/clc.22631 PMC6490535 28244595

[8] Soumarová R , Rusinová L . Cardiotoxicity of breast cancer radiotherapy – overview of current results. Rep Pract Oncol Radiother. 2020;25:182‐186. doi:10.1016/j.rpor.2019.12.008 PMC6994274 32021574

[9] Darby SC , Ewertz M , McGale P , et al. Risk of ischemic heart disease in women after radiotherapy for breast cancer. N Engl J Med. 2013;368(11):987‐998. doi:10.1056/NEJMoa1209825 23484825

[10] Bogaard VABVD , Ta BDP , Schaaf AVD , et al. Validation and modification of a prediction model for acute cardiac events in patients with breast cancer treated with radiotherapy based on three‐dimensional dose distributions to cardiac substructures. J Clin Oncol. 2017;35(11):1171‐1178. doi:10.1200/JCO.2016.69.8480 PMC5455600 28095159

[11] Talebi A , Bitarafan‐Rajabi A , Alizadeh‐asl A , et al. Machine learning based radiomics model to predict radiotherapy induced cardiotoxicity in breast cancer. J Appl Clin Med Phys. 2025;26(4):e14614. doi:10.1002/acm2.14614 PMC11969081 39704607

[12] Dincer S , Akmansu M , Akyol O . Machine learning modeling of cancer treatment‐related cardiac events in breast cancer: utilizing dosiomics and radiomics. Front Oncol. 2025;15:1557382. doi:10.3389/fonc.2025.1557382 PMC12408287 40919168

[13] Khan SS , Coresh J , Pencina MJ , et al. Novel prediction equations for absolute risk assessment of total cardiovascular disease incorporating cardiovascular‐kidney‐metabolic health: a scientific statement from the American Heart Association. Circulation. 2023;148(24):1982‐2004. doi:10.1161/CIR.0000000000001191 37947094

[14] Bazan M , Gutiérrez‐Villamil C , Peix A , et al. Cardio‐oncology in Latin America and the Caribbean. Current state. ecancermedicalscience. 2025;19:2014. doi:10.3332/ecancer.2025.2014 PMC12812807 41561514

[15] Birn AE , Pillay Y , Holtz TH . Textbook of Global Health. 4th ed. Oxford University Press; 2017.

[16] Osei Baah F , Sharda S , Davidow K , et al. Social determinants of health in cardio‐oncology: multi‐level strategies to overcome disparities in care: JACC: CardioOncology state‐of‐the‐art review. JACC CardioOncol. 2024;6(3):331‐346. doi:10.1016/j.jaccao.2024.02.009 PMC11229550 38983377

[17] Merino T , Pinto MP , Orellana MP , et al. Multimodal assessment of acute cardiac toxicity induced by thoracic radiotherapy in cancer patients. Study protocol. BMC Cancer. 2021;21(1):1114. doi:10.1186/s12885-021-08823-3 PMC8524888 34663256

[18] Duane F , Aznar MC , Bartlett F , et al. A cardiac contouring atlas for radiotherapy. Radiother Oncol. 2017;122:416‐422. doi:10.1016/j.radonc.2017.01.008 PMC5356506 28233564

[19] National Cancer Institute . Common terminology criteria for adverse events (CTCAE), version 5.0. U.S. Department of Health and Human Services, National Institutes of Health, National Cancer Institute; 2017. Available from: https://ctep.cancer.gov/protocolDevelopment/electronic_applications/ctc.htm

[20] Plana JC , Galderisi M , Barac A , et al. Expert consensus for multimodality imaging evaluation of adult patients during and after cancer therapy. J Am Soc Echocardiogr. 2014;27(9):911‐939. doi:10.1016/j.echo.2014.07.012 25172399

[21] Vittinghoff E , McCulloch CE . Relaxing the rule of ten events per variable in logistic and Cox regression. Am J Epidemiol. 2007;165(6):710‐718. 10.1093/aje/kwk052 17182981

[22] Khan SS , Matsushita K , Sang Y , et al. Development and validation of the american heart association's PREVENT equations. Circulation. 2024;149(6):430‐449. doi:10.1161/CIRCULATIONAHA.123.067626 PMC10910659 37947085

[23] Jolliffe IT , Cadima J . Principal component analysis: a review and recent developments. Philos Trans R Soc A. 2016;374(2065):20150202. 10.1098/rsta.2015.0202 PMC4792409 26953178

[24] James G , Witten D , Hastie T , Tibshirani R . An Introduction to Statistical Learning with Applications in R. 2nd edition. Springer; 2021. Available from: http://www.springer.com/series/417

[25] Rand WM . Objective criteria for the evaluation of clustering methods. J Am Stat Assoc. 1971;66(336):846‐850.

[26] Rousseeuw PJ . Silhouettes: a graphical aid to the interpretation and validation of cluster analysis. J Comput Appl Math. 1987;20:53‐65. doi:10.1016/0377-0427(87)90125-7

[27] Firth D . Bias reduction of maximum likelihood estimates. Biometrika. 1993;80(1):27‐38.

[28] Heinze G , Schemper M . A solution to the problem of separation in logistic regression. Stat Med. 2002;21(16):2409‐2419. 10.1002/sim.1047 12210625

[29] Efron B , Tibshirani R . Improvements on cross‐validation: the .632+ bootstrap method. J Am Stat Assoc. 1997;92(438):548‐560.

[30] Brier GW . Verification of forecasts expressed in terms of probability. Mon Weather Rev. 1950;78(1):1‐3.

[31] Hosmer DW , Lemeshow S . Goodness of fit tests for the multiple logistic regression model. Commun Stat Theory Methods. 1980;9(10):1043‐1069.

[32] Vickers AJ , Elkin EB . Decision curve analysis: a novel method for evaluating prediction models. Med Decis Making. 2006;26(6):565‐574. 10.1177/0272989X06295361 PMC2577036 17099194

[33] Riley RD , Snell KIE , Ensor J , et al. Minimum sample size for developing a multivariable prediction model: part II ‐ binary and time‐to‐event outcomes. Statistics in Medicine. 2019;38:1276‐1296. doi:10.1002/sim.7992 PMC6519266 30357870

[34] Yang WC , Chen YH , Wang CC , et al. Evaluation of cardiotoxicity following breast cancer irradiation in an Asian cohort in real‐world settings. Breast. 2025;84:104581. doi:10.1016/j.breast.2025.104581 PMC12495232 40991981

[35] Cuculich PS , Schill MR , Kashani R , et al. Noninvasive cardiac radiation for ablation of ventricular tachycardia. N Engl J Med. 2017;377(24):2325‐2336. doi:10.1056/NEJMoa1613773 PMC5764179 29236642

[36] Zei PC , Mak R . Noninvasive stereotactic radioablation for ventricular tachycardia: ENCORE‐VT (EP‐guided noninvasive cardiac radioablation): is the sequel as good as the original? Circulation. 2019;139(3):322‐324. doi:10.1161/CIRCULATIONAHA.118.038285 30586783

[37] Lyon AR , López‐Fernández T , Couch LS , et al. 2022 ESC guidelines on cardio‐oncology developed in collaboration with the European Hematology Association (EHA), the European Society for Therapeutic Radiology and Oncology (ESTRO) and the International Cardio‐Oncology Society (IC‐OS). Eur Heart J. 2022;43(41):4229‐4361. doi:10.1093/eurheartj/ehac244 36017568

