# Supplementary material for: Machine learning predictors of post‐radiotherapy cardiotoxicity in a Latin American breast cancer cohort: Clinical risk factors outperform cardiac dosimetry
Source: J Appl Clin Med Phys. 2026 Oct 14;27(10):e70862. doi: 10.1002/acm2.70862 (PMC13669535; doi:10.1002/acm2.70862)
Supplement: Supplementary file 1 [file ACM2-27-e70862-s001.pdf]

Supplementary Material

Machine Learning Predictors of Post-Radiotherapy Cardiotoxicity in a Latin American Breast Cancer Cohort: Clinical Risk Factors Outperform Cardiac Dosimetry

Víctor Aguirre-Bravo, Rodrigo El Far, María Herrera Ruiz, Sophia Gálvez Santelices, Pablo Munoz-Schuffenegger, Tomás Merino, Sergio Diez Domingo, Marcelo E. Andia

Abbreviations: CDT, post-RT cardiotoxicity (CTCAE v5.0 ≥Grade 1, excluding the non-specific term *"Cardiac disorders – Other, specify"*; $n = 10$, 23.8%); RT, radiotherapy; DVH, dose-volume histogram; IQR, interquartile range; CTCAE, Common Terminology Criteria for Adverse Events; OR, odds ratio; CI, confidence interval; AUC, area under the ROC curve; LV, left ventricle; RV, right ventricle; LA, left atrium; RA, right atrium; LAD, left anterior descending artery; D_{mean}, mean dose; V_x, percentage of volume receiving $\geq x$ Gy; 3D-CRT, three-dimensional conformal radiotherapy; IMRT, intensity-modulated radiotherapy; PCA, principal component analysis; K-PCA, kernel PCA; $\mathrm{CDT_A}$, any Grade ≥1 (sensitivity); $\mathrm{CDT_B}$, Grade ≥2 (sensitivity).

Table S1. Post-RT Cardiovascular Events by CTCAE v5.0 Term and Grade

All CTCAE-graded post-RT cardiovascular events recorded in the cohort, classified according to CTCAE version 5.0. For each term, the number of patients affected and the percentage of the full cohort ($n = 42$) are reported; cases with more than one CTCAE term are counted once per term. The first row—*"Cardiac disorders – Other, specify"*—is **excluded from the primary outcome definition** (Section 2.4 of the main text) and is shown here for completeness. The remaining terms define the 10 CDT+ patients.

**CTCAE v5.0 Term**	**Grade**	**n patients**	**% cohort**
*Cardiac disorders — Other, specify**	*1 (Mild)*	*7*	*16.7*
Sinus bradycardia	1 (Mild)	2	4.8
Aortic valve disease	1 (Mild)	2	4.8
Heart failure	1 (Mild)	1	2.4
Sinus tachycardia	1 (Mild)	1	2.4
Atrioventricular block, first degree	1 (Mild)	1	2.4
Mitral valve disease	2 (Moderate)	1	2.4
Myocardial infarction	2 (Moderate)	1	2.4
Left ventricular systolic dysfunction	3 (Severe)	1	2.4
Atrial fibrillation	3 (Severe)	1	2.4
Atrioventricular block, complete	3 (Severe)	1	2.4
Conduction disorder	3 (Severe)	1	2.4
Myocardial infarction	5 (Death)	1	2.4

Grade according to CTCAE v5.0 (2017). Patients may present more than one CTCAE term; n patients counts distinct individuals per term. Median time from end of RT to the first qualifying event in CDT+ patients: 48.4 months [IQR: 21.6–65.5].

* Excluded from the primary outcome. On case-by-case review these 11 events were incidental findings—mildly enlarged cardiac silhouette on chest radiography ($n = 4$), calcified atheromatosis on computed tomography ($n = 2$), mild ventricular hypertrophy on echocardiography ($n = 2$), non-specific repolarization changes on electrocardiography ($n = 2$) and rare atrial extrasystoles on Holter monitoring ($n = 1$)—and 6 of the 11 arose from imaging requested for an unrelated indication. Two of these 7 patients had no other recorded event and are therefore CDT−.

Figure S1a. Post-RT Cardiovascular Events by CTCAE v5.0 Term and Grade

Horizontal stacked bar chart showing the number of patients affected by each CTCAE v5.0 term, stratified by event grade. Bars are colour-coded by severity grade (blue: Grade 1; yellow: Grade 2; orange: Grade 3; purple: Grade 5). Arrhythmic and conduction disorders are the most frequent category; Grade 1 events predominate. The non-specific term (marked with ‡) is excluded from the primary outcome definition.

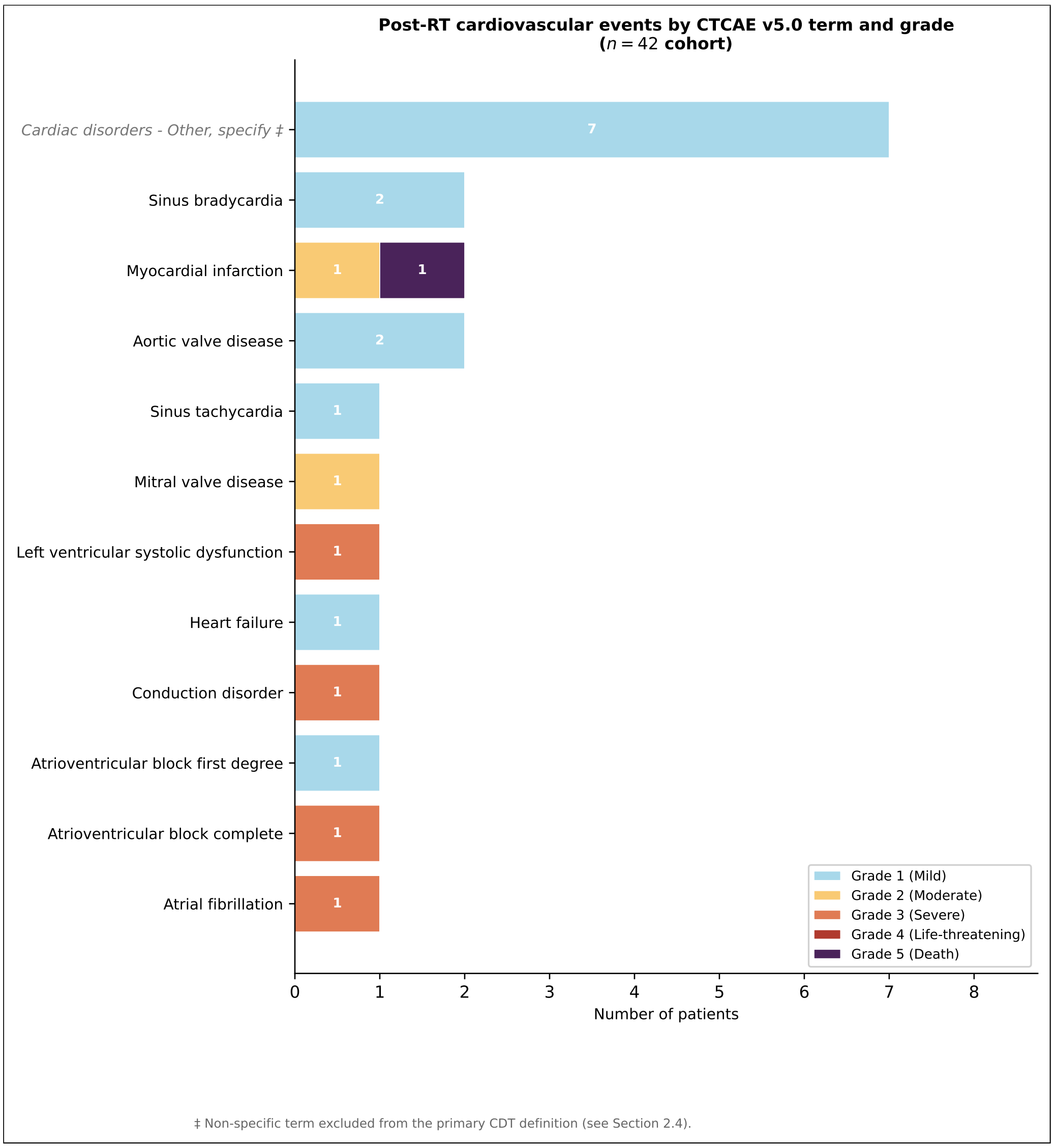

Figure S1a. Post-RT cardiovascular events by CTCAE v5.0 term and grade ($n = 42$ cohort).

Figure S1b. Timeline of Post-RT Cardiovascular Events

Timeline of post-RT cardiovascular events with **one row per patient** (P01–P12, ordered by time to the first qualifying event). Because a patient may experience more than one event, a single row can carry several markers; this figure has been redrawn from the previous submission so that repeated events in the same patient can no longer be read as separate patients. Filled circles with a heavy dark outline mark each patient's *first qualifying event*—the quantity entering the median time-to-event and the event counts of Section 3.2—and smaller white-rimmed circles mark subsequent events in the same patient. Colour denotes CTCAE v5.0 grade. Open squares mark events coded under the non-specific term *"Cardiac disorders – Other, specify"*: these are displayed for transparency but are *not* counted as cardiac events under the primary CDT definition (Section 2.4), and patients whose only post-RT findings were non-specific are labelled in italic grey and classified CDT−. The shaded band and dashed vertical lines indicate the 6- and 12-month landmarks.

Twenty-nine CTCAE-graded events occurred in 12 patients, of whom 10 met the primary CDT definition. Their median time to the first qualifying event was 48.4 months [IQR: 21.6–65.5], and two of the ten had that event within 12 months (2.7 and 6.9 months); the second marker inside the 12-month band on row P03 is a recurrent myocardial infarction in that same patient at 7.8 months, not a third patient. The earliest event in the cohort (2.7 months, Grade 1 aortic valve disease) is a qualifying event retained in the primary outcome, whereas the two earliest non-specific findings occurred at 6.2 and 7.6 months in patients classified CDT−.

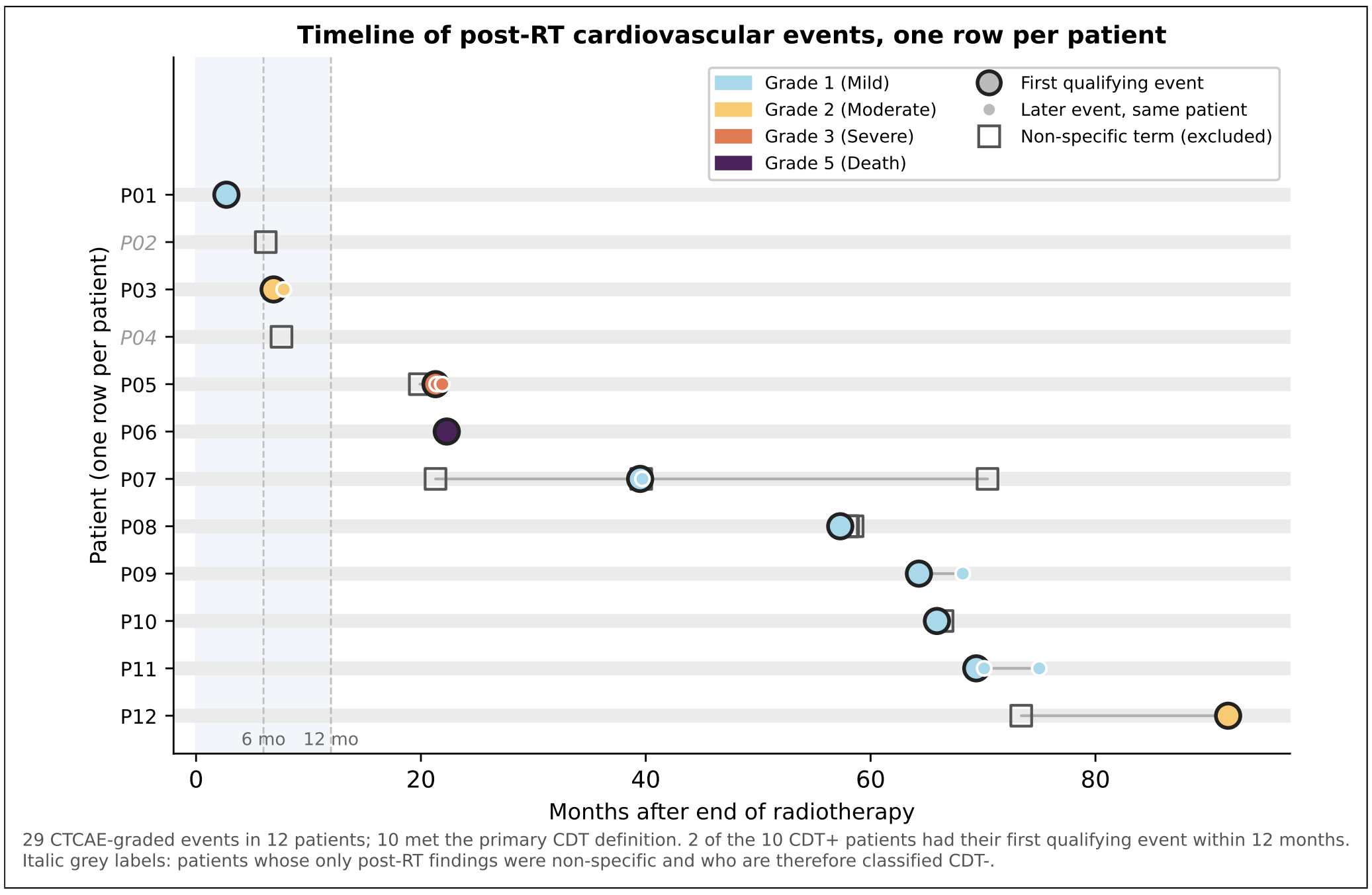

Figure S1b. Timeline of post-RT cardiovascular events, one row per patient, by CTCAE grade and outcome classification.

Figure S1c. Pre-RT versus Post-RT Cardiovascular Events by CTCAE v5.0 Term

Grouped bar chart comparing the number of patients with each CTCAE v5.0 term before (pre-RT, blue) and after (post-RT, orange) radiotherapy. The comparison illustrates which event types are newly incident post-RT versus those reflecting pre-existing disease.

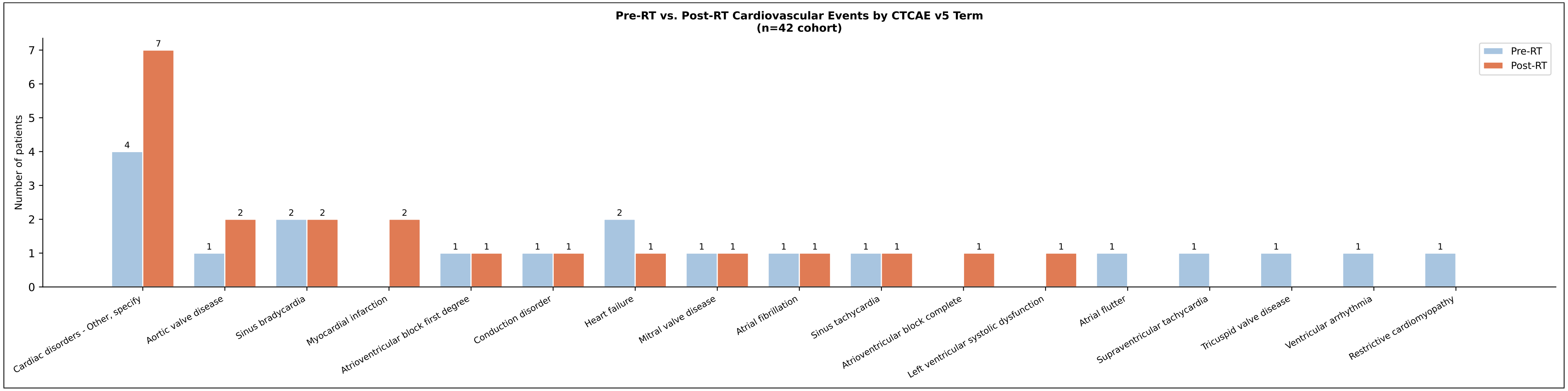

Figure S1c. Pre-RT vs. post-RT cardiovascular events by CTCAE v5.0 term ($n = 42$ cohort).

Table S2. DVH Parameters by CDT Group (CDT+ vs. CDT−)

Dose-volume histogram parameters for all seven cardiac substructures, compared between patients with (CDT+) and without (CDT−) post-RT cardiotoxicity. Values are median [IQR]. Statistical comparisons performed with Mann-Whitney U test (two-sided). CDT+: post-RT cardiotoxicity per the primary definition (Section 2.4). All p-values > 0.05. $n = 42$.

**Parameter**	**CDT+** ($n = 10$)	**CDT−** ($n = 32$)	p**-value**
Heart			
D_{mean} (Gy)	2.73 [2.58–3.08]	2.41 [1.72–4.17]	0.757
D_{max} (Gy)	36.25 [14.38–40.58]	28.00 [6.23–39.40]	0.658
V_5 (%)	6.94 [6.15–10.51]	6.52 [0.73–13.54]	0.894
V_{10} (%)	3.99 [0.49–5.37]	2.39 [0.00–4.95]	0.621
V_{20} (%)	1.20 [0.19–3.09]	0.23 [0.00–2.74]	0.588
V_{30} (%)	0.34 [0.03–2.03]	0.00 [0.00–1.35]	0.466
V_{40} (%)	0.00 [0.00–0.12]	0.00 [0.00–0.00]	0.364
LAD (left anterior descending artery)			
D_{mean} (Gy)	6.65 [2.21–21.32]	10.31 [1.50–17.55]	1.000
D_{max} (Gy)	18.60 [2.70–40.90]	31.35 [2.80–39.65]	0.838
V_5 (%)	61.00 [0.00–69.48]	64.74 [0.00–79.04]	0.487
V_{10} (%)	8.88 [0.00–58.39]	36.22 [0.00–64.83]	0.570
V_{20} (%)	0.00 [0.00–46.97]	5.26 [0.00–44.23]	0.742
V_{30} (%)	0.00 [0.00–36.30]	0.19 [0.00–27.95]	0.881
Left ventricle (LV)			
D_{mean} (Gy)	3.57 [2.19–4.40]	3.85 [0.91–5.08]	0.353
D_{max} (Gy)	33.20 [4.10–37.40]	27.95 [2.42–39.55]	0.560
V_5 (%)	14.60 [0.00–15.43]	14.10 [0.00–24.08]	0.236
V_{10} (%)	4.62 [0.00–9.34]	4.65 [0.00–10.18]	0.615
V_{20} (%)	0.96 [0.00–6.38]	0.50 [0.00–5.36]	0.934
Right ventricle (RV)			
D_{mean} (Gy)	2.53 [2.02–3.98]	2.21 [1.51–4.07]	1.000
V_5 (%)	6.36 [1.14–15.92]	3.78 [0.00–17.98]	0.836
Left atrium			
D_{mean} (Gy)	0.86 [0.80–1.98]	1.02 [0.77–2.33]	0.539
V_5 (%)	0.00 [0.00–0.00]	0.00 [0.00–0.00]	0.927
Right atrium			
D_{mean} (Gy)	0.85 [0.81–1.45]	1.32 [0.83–2.40]	0.694
V_5 (%)	0.00 [0.00–0.00]	0.00 [0.00–0.19]	0.872
Aorta			
D_{mean} (Gy)	0.92 [0.62–1.27]	0.93 [0.72–1.67]	0.518
V_5 (%)	0.00 [0.00–0.00]	0.00 [0.00–4.00]	0.542

Bold: $p < 0.05$. †: $p < 0.10$ (trend). Mann-Whitney U, two-sided.

Table S3. DVH Parameters by Delivery Technique (3D-CRT vs. IMRT)

Dose-volume histogram parameters for all seven cardiac substructures, compared between 3D conformal radiotherapy (3D-CRT, $n = 33$) and intensity-modulated radiotherapy (IMRT, $n = 9$). Values are median [IQR]; comparisons by two-sided Mann-Whitney U test. separately in Section 3.2, as that case (right-sided disease, 30 Gy in 5 fractions) is not comparable to the IMRT group; one patient with missing technique is also excluded. The effect size $r = |Z|/\sqrt{N}$ is reported alongside the p-value, since group sizes are unequal and p-values alone do not convey magnitude. Bold values: $p < 0.05$. †: $p < 0.10$ (trend).

Parameter	3D-CRT ($n = 33$)	IMRT ($n = 9$)	p-value	r
Heart				
D_{mean} (Gy)	2.29 [1.68–2.85]	4.26 [3.60–4.92]	**0.009**	0.40
D_{max} (Gy)	37.20 [5.70–39.40]	23.90 [21.60–48.90]	0.646	0.07
V_5 (%)	6.06 [0.18–9.08]	26.56 [12.48–31.74]	**0.001**	0.51
V_{10} (%)	2.44 [0.00–4.54]	3.71 [2.12–5.08]	0.202	0.20
V_{20} (%)	0.82 [0.00–3.09]	0.12 [0.05–1.13]	0.791	0.04
V_{30} (%)	0.30 [0.00–2.00]	0.00 [0.00–0.50]	0.385	0.13
V_{40} (%)	0.00 [0.00–0.00]	0.00 [0.00–0.15]	0.489	0.09
LAD (left anterior descending artery)				
D_{mean} (Gy)	6.65 [1.40–19.81]	11.84 [7.72–17.19]	0.617	0.08
D_{max} (Gy)	30.90 [2.50–40.10]	27.85 [14.35–40.20]	0.610	0.08
V_5 (%)	46.23 [0.00–72.09]	89.94 [51.85–93.36]	**0.032**	0.33
V_{10} (%)	15.38 [0.00–60.55]	55.53 [25.26–62.63]	0.301	0.16
V_{20} (%)	3.40 [0.00–50.97]	8.99 [0.00–33.77]	0.945	0.01
V_{30} (%)	0.06 [0.00–35.36]	0.16 [0.00–13.17]	0.768	0.05
Left ventricle (LV)				
D_{mean} (Gy)	3.46 [0.87–4.66]	4.55 [3.22–5.40]	0.176	0.22
D_{max} (Gy)	37.40 [2.20–39.60]	18.10 [10.70–29.95]	0.730	0.06
V_5 (%)	12.11 [0.00–16.58]	25.80 [9.43–45.11]	0.070†	0.28
V_{10} (%)	5.07 [0.00–10.03]	3.25 [0.36–5.13]	0.879	0.03
V_{20} (%)	2.15 [0.00–6.57]	0.00 [0.00–0.88]	0.246	0.17
Right ventricle (RV)				
D_{mean} (Gy)	2.02 [1.51–3.06]	4.65 [4.12–5.98]	**0.004**	0.43
V_5 (%)	2.68 [0.00–9.83]	35.06 [20.51–65.62]	**0.001**	0.50
Left atrium				
D_{mean} (Gy)	0.86 [0.74–1.26]	2.54 [1.95–3.56]	**0.005**	0.44
V_5 (%)	0.00 [0.00–0.00]	0.00 [0.00–5.75]	**0.004**	0.24
Right atrium				
D_{mean} (Gy)	1.00 [0.78–1.80]	2.76 [1.83–5.11]	**0.011**	0.40
V_5 (%)	0.00 [0.00–0.00]	1.03 [0.00–31.33]	0.055†	0.24
Aorta				
D_{mean} (Gy)	0.89 [0.65–1.25]	3.72 [2.58–5.36]	**0.007**	0.41
V_5 (%)	0.00 [0.00–0.00]	19.39 [11.19–42.18]	**0.001**	0.45

Bold: $p < 0.05$. †: $p < 0.10$ (trend). Mann-Whitney U, two-sided. r: effect size.

Table S4. Cardiac Substructure Mean Dose by CDT Group

Mean dose (D_{mean}, Gy) for all seven cardiac substructures in CDT+ and CDT− patients. No statistically significant differences were observed (Mann-Whitney U, all $p > 0.05$), confirming consistently low radiation exposure across the entire cohort.

**Cardiac structure**	**CDT+** **(n = 10)** Median [IQR] Gy	**CDT−** **(n = 32)** Median [IQR] Gy	***p*-value**
Heart	2.73 [2.58–3.08]	2.41 [1.72–4.17]	0.757
LAD	6.65 [2.21–21.32]	10.31 [1.50–17.55]	1.000
Left Ventricle	3.57 [2.19–4.40]	3.85 [0.91–5.08]	0.353
Right Ventricle	2.53 [2.02–3.98]	2.21 [1.51–4.07]	1.000
Left Atrium	0.86 [0.80–1.98]	1.02 [0.77–2.33]	0.539
Right Atrium	0.85 [0.81–1.45]	1.32 [0.83–2.40]	0.694
Aorta	0.92 [0.62–1.27]	0.93 [0.72–1.67]	0.518

Mann-Whitney U, two-sided. All $p > 0.05$.

Figure S2a. DVH Curves by CDT Group

Mean dose-volume histogram curves for the whole heart, left ventricle (LV), and LAD, stratified by CDT outcome. Solid lines indicate median DVH; shaded bands indicate IQR. No meaningful separation between CDT+ and CDT− curves is observed, consistent with the absence of statistically significant DVH differences between groups.

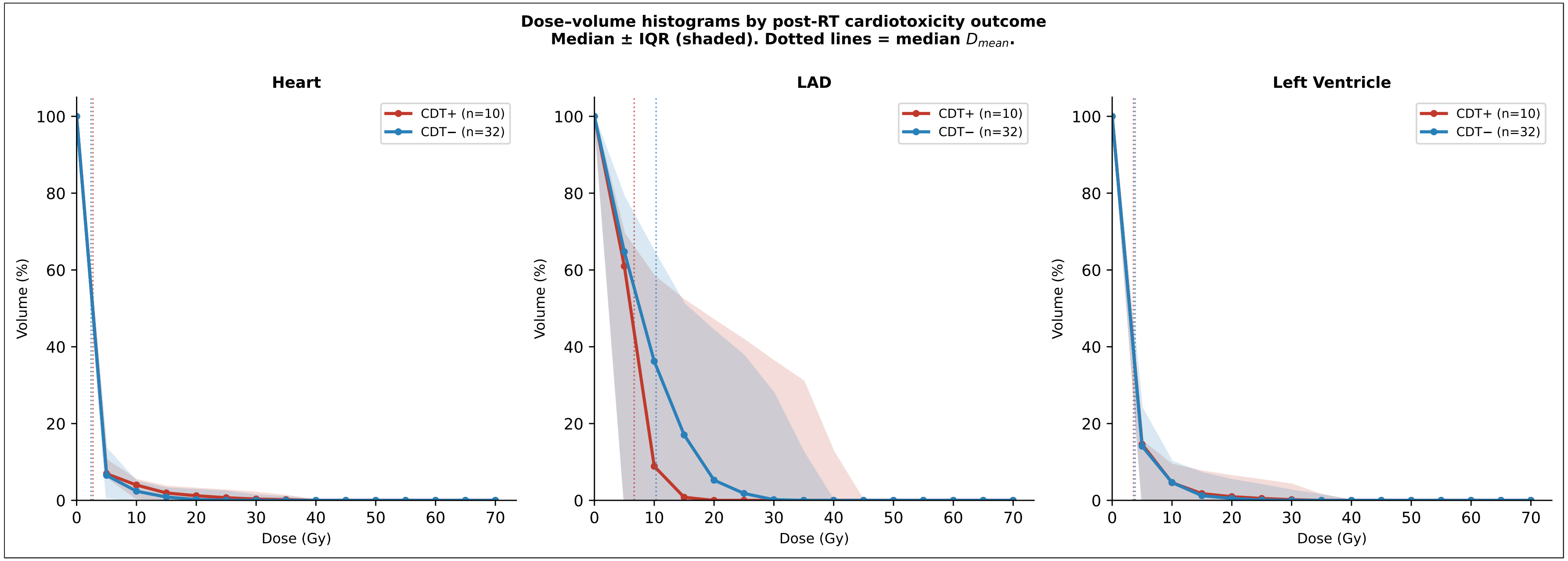

Figure S2a. Mean DVH curves for Heart, LV, and LAD by CDT group. Shaded area: IQR.

Figure S2b. DVH Curves by Radiotherapy Technique

Mean dose-volume histogram curves comparing 3D conformal radiotherapy (3D-CRT, $n = 33$) and intensity-modulated radiotherapy (IMRT, $n = 9$) for the whole heart. The shift in the IMRT curves toward higher low-dose volumes illustrates the characteristic low-dose bath effect of IMRT optimization.

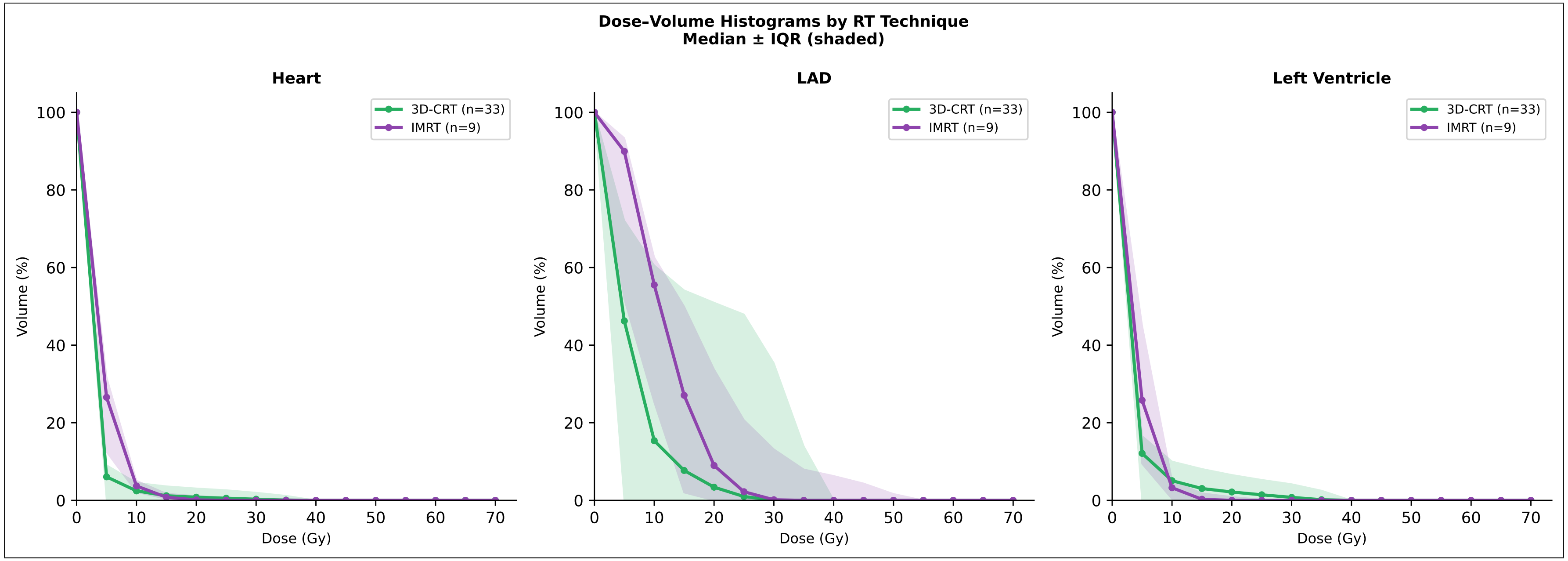

Figure S2b. Mean DVH curves by RT technique (3D-CRT vs. IMRT) for the whole heart.

Figure S2c. Cardiac Substructure Mean Dose by CDT Group

Dot plot with IQR bars showing the mean dose (D_{mean}, Gy) for each cardiac substructure in CDT+ (filled markers) and CDT− (open markers) patients. All substructure doses were consistently low in both groups, with no significant between-group differences (all Mann-Whitney U $p > 0.05$).

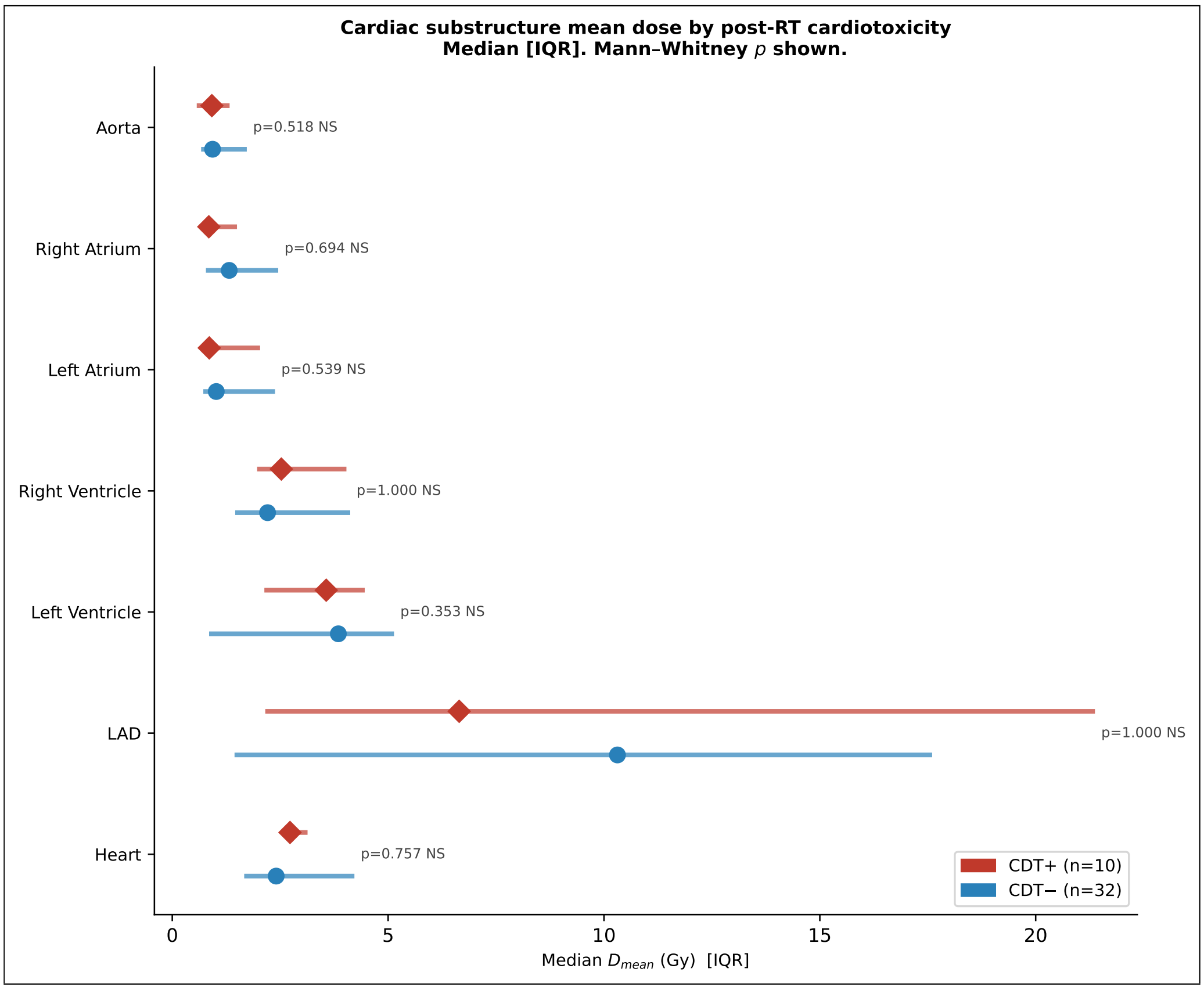

Figure S2c. Cardiac substructure D_{mean} (Gy) by CDT group. Markers: median; bars: IQR.

Table S5. DVH Parameters by CDT Group, Restricted to Left-Sided RT

Sensitivity analysis restricting the DVH comparison between CDT+ and CDT− patients to the subgroup treated with left-sided RT (n = 25: 6 CDT+, 19 CDT−; 1 bilateral case and 16 right-sided cases excluded), since right-sided RT typically delivers near-zero cardiac dose and may dilute a dose-effect signal specific to the left-sided subgroup. Values are median [IQR]. Statistical comparisons performed with Mann-Whitney U test (two-sided); not corrected for multiple comparisons.

**Parameter**	**CDT+** ($n = 5$)	**CDT−** ($n = 20$)	p**-value**
Heart D_{mean} (Gy)	2.71 [2.60–2.74]	3.22 [2.46–4.60]	0.447
Heart V_5 (%)	7.41 [6.48–9.08]	11.69 [6.64–19.32]	0.303
LAD D_{mean} (Gy)	21.32 [16.92–21.75]	15.90 [11.97–23.20]	0.575
LV D_{mean} (Gy)	4.40 [3.73–4.65]	4.92 [3.97–5.40]	0.169
LV V_5 (%)	15.43 [15.25–16.08]	18.92 [16.16–26.34]	0.071†

Bold: $p < 0.05$. †: $p < 0.10$ (trend). Mann-Whitney U, two-sided; exploratory, not corrected for multiple comparisons (5 metrics tested).

Even within the left-sided subgroup, no DVH metric was higher in CDT+ than in CDT−; LV V_5 was nominally *lower* in CDT+ ($p = 0.030$), opposite to the direction expected under a direct dose-effect hypothesis. This pattern is consistent with the cardioprotective indication-bias hypothesis discussed in the main text (Discussion).

Abbreviations: CDT, post-RT cardiotoxicity; RT, radiotherapy; LV, left ventricle; LAD, left anterior descending artery; D_{mean}, mean dose; V_5, percentage of volume receiving ≥ 5 Gy; IQR, interquartile range.

Table S6. Detailed Univariate Odds Ratios (Bootstrap 95% CI)

Univariate analysis for the primary outcome (Post-RT Cardiotoxicity) and the secondary outcome (Post-RT CVD Events). OR values were estimated using Firth penalized logistic regression to account for the low number of events ($n = 42$).

	**Post-RT Cardiotoxicity**		**Post-RT CVD Events**	
**Variable**	**OR [95% CI]**	***p*-value**	**OR [95% CI]**	***p*-value**
Clinical Variables				
Age at RT[c]	1.05 [1.00–1.13]	0.089	1.03 [0.99–1.09]	0.199
Pre-RT Cardiac Disease	6.50 [1.40–40.24]	0.020*	5.50 [1.40–31.96]	0.032*
Hypertension	2.20 [0.48–9.63]	0.451	1.75 [0.48–6.59]	0.511
Previous CTx (prior cancer)[a]	7.75 [0.62–44.73]	0.136	3.57 [0.34–19.67]	0.547
Chemotherapy (current episode)[b]	0.19 [0.03–0.90]	0.071	0.51 [0.14–1.84]	0.354
Dosimetric Variables[c]				
Heart D_{mean} (Gy)	1.02 [0.62–1.55]	0.757	1.09 [0.60–1.39]	0.866
Heart V_5 (%)	1.00 [0.94–1.06]	0.894	1.01 [0.94–1.05]	0.907
LAD D_{mean} (Gy)	1.00 [0.92–1.08]	1.000	0.99 [0.91–1.06]	0.695

Note: * indicates $p < 0.05$. CI estimated via bootstrap resampling (2,000 replicates); p from Fisher's exact test (binary predictors) or Mann-Whitney U (continuous).

[a] Chemotherapy administered for a *previous* malignancy, before the index breast cancer. Only 3 of 42 patients were exposed (24 records were blank; in 23 of these the patient had no previous cancer, so the field was not applicable and was coded as unexposed, leaving one genuinely unknown). Estimates are correspondingly unstable and should not be interpreted as evidence of effect.

[b] Chemotherapy for the *index* breast cancer (neoadjuvant, adjuvant or concomitant; 20 of 42 exposed, 3 unrecorded). The inverse association most likely reflects confounding by indication, as patients selected for chemotherapy were younger and had less baseline cardiovascular comorbidity.

[c] Odds ratio per unit increase (one year, 1 Gy or 1%). The dose-volume estimates in this table are computed from the same DVH extraction used throughout the manuscript; in the originally submitted version they were inadvertently derived from a second, superseded dose-volume derivation, which is corrected here. None of them is statistically significant under either derivation.

Abbreviations: OR, odds ratio; CI, confidence interval; RT, radiotherapy; CVD Events, post-RT cardiovascular events; LAD, left anterior descending artery; D_{mean}, mean dose; V_5, percentage of heart volume receiving ≥5 Gy.

Figure S3. Collinearity Analysis of Dosimetric Parameters

Spearman correlation matrix of the pre-specified dosimetric variables. High collinearity ($\rho > 0.8$) is observed among heart and left ventricular (LV) metrics, justifying the use of representative predictors in the combined models.

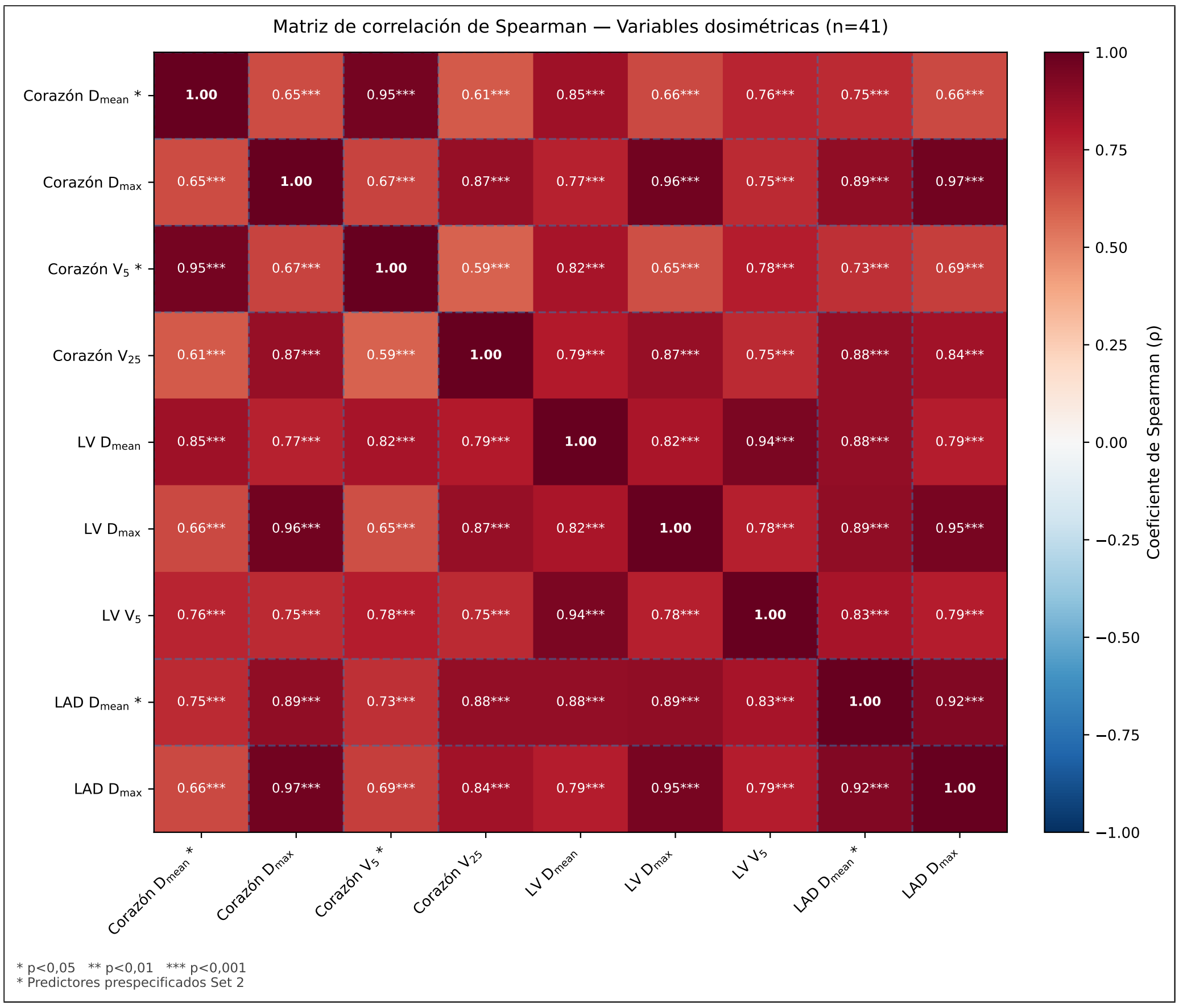

Figure S3. Spearman correlation matrix of dosimetric parameters ($n = 42$).

Table S7. K-means Cluster Validation: Silhouette and Davies-Bouldin Scores

Table S7. Silhouette coefficient, Davies-Bouldin index, and within-cluster inertia for K-means clustering ($k = 2–5$) applied to the first two principal components of each variable set ($n = 42$). Higher silhouette and lower Davies-Bouldin indicate better cluster separation. K-means++ initialization, 10 restarts, random seed 42.

Variable Set	k	Silhouette	Davies-Bouldin	Inertia
Set 1 — Clinical	2	0.466	0.905	66.56
	3	0.518	0.672	32.67
	4	0.497	0.761	23.36
	5	0.549	0.637	15.50
Set 2 — Dosimetric [a]	**2**	**0.770**	**0.153**	**64.03**
	3	0.543	0.469	24.49
	4	0.609	0.487	11.76
	5	0.558	0.508	7.92

Bold row indicates the selected k for each set. For Set 2, $k = 2$ achieves the highest silhouette (0.770), providing strong geometric justification. For Set 1, $k = 2$ was selected based on clinical interpretability (binary CDT outcome) despite $k = 3$ yielding a slightly higher geometric silhouette; the purpose of the clustering is hypothesis-testing against a known binary outcome label rather than unsupervised discovery.

[a] Set 2 was clustered on the 41 patients with complete dosimetric data (one patient lacked LAD dose; see Table 3 of the main text).

Abbreviations: CDT, post-RT cardiotoxicity; PCA, principal component analysis.

Table S8. Sensitivity Analysis: Key Predictors Under Alternative CDT Definitions

Table S8. Univariate association of key clinical predictors with cardiotoxicity under three outcome definitions: the primary definition (Grade ≥1 restricted to specific CTCAE terms) and the two pre-specified sensitivity definitions. Fisher's exact test for binary predictors; Mann-Whitney U test for continuous predictors.

**Predictor**	**Primary: Grade ≥1, specific terms** ($n = 10$, 23.8%)	**CDT_A: any Grade ≥1** ($n = 12$, 28.6%)	**CDT_B: Grade ≥2** ($n = 4$, 9.5%)
Pre-existing cardiovascular disease			
OR (crude)	6.50	7.00	$\infty^{\dagger}$
p-value	**0.020**	**0.019**	**0.004**
Event rate: exposed vs. unexposed	6/12 vs. 4/30	7/12 vs. 5/30	4/12 vs. 0/30
Hypertension			
OR (crude)	2.20	3.85	1.92
p-value	0.451	$0.078^{\ddagger}$	0.608
Age at RT (years)			
Median CDT− vs. CDT+	53.0 vs. 63.5	51.5 vs. 63.5	59.0 vs. 59.5
p-value	$0.089^{\ddagger}$	$0.056^{\ddagger}$	0.492
Previous CTx (prior cancer)§			
OR (crude)	7.75	5.80	37.00
p-value	0.136	0.192	**0.020**
Event rate: exposed vs. unexposed	2/3 vs. 8/39	2/3 vs. 10/39	2/3 vs. 2/39
Chemotherapy (current episode)¶			
OR (crude)	0.19	0.25	0.00
p-value	$0.071^{\ddagger}$	$0.091^{\ddagger}$	0.109
Event rate: exposed vs. unexposed	2/20 vs. 8/22	3/20 vs. 9/22	0/20 vs. 4/22

† Undefined (infinite) OR: all 4 Grade ≥2 events occurred in patients with pre-existing cardiovascular disease (0 events in 30 patients without pre-existing CVD). Fisher's exact $p = 0.004$.

‡ $p < 0.10$, trend.

§ Only 3 of 42 patients were exposed; the apparently large odds ratio under CDT_B rests on 2 of those 3 patients and is statistically unstable. Reported for completeness, not as a finding.

¶ Chemotherapy for the index breast cancer. The consistently inverse association is most plausibly confounding by indication rather than a protective effect.

A fourth, post-hoc definition excluding the 13 events flagged in the clinical record as related to a pre-existing condition yields $n = 7$ (16.7%) and dissolves the association with pre-existing cardiovascular disease (OR 2.17, $p = 0.387$). It is reported in the Discussion but not used, because it is circular by construction: it preferentially removes events from the exposed group.

Abbreviations: CDT, post-RT cardiotoxicity; OR, crude odds ratio from 2×2 table; CTx, chemotherapy; CVD, cardiovascular disease.

Table S9. Adjusted Rand Index of K-means Clustering

Adjusted Rand Index (ARI) of the K-means clustering ($k = 2$) applied to the first two principal components (PCA) for each variable set and outcome.

**Outcome**	**Variable Set**	**ARI**
Post-RT CDT	Set 1 Clinical	0.045
Post-RT CDT	Set 2 Dosimetric	−0.032
Post-RT CVD Events	Set 1 Clinical	0.011
Post-RT CVD Events	Set 2 Dosimetric	0.030

Abbreviations: ARI, Adjusted Rand Index; CDT, post-RT CDT (primary outcome); CVD Events, post-RT cardiovascular events (secondary outcome); PCA, principal component analysis.

Table S10. Firth Regression Stratified by AHA PREVENT Category

Firth penalized logistic regression results stratified by AHA PREVENT categories.

Category	OR	95% CI	*p-value*
**Post-RT CDT**			
Low ($< 3\%$)	1.00	—	—
Borderline+Intermediate	3.28	0.53–20.21	0.200
High ($\geq 10\%$)	2.94	0.48–17.79	0.241
**Post-RT CVD Events**			
Low ($< 3\%$)	1.00	—	—
Borderline+Intermediate	4.09	0.84–19.99	0.082†
High ($\geq 10\%$)	1.94	0.41–9.27	0.406

†$p < 0.10$; $*p < 0.05$. Cochran-Armitage trend test for CDT: $z = 1.274$, $p = 0.203$ (not significant).
Abbreviations: OR, odds ratio; CI, confidence interval; LRT, Likelihood Ratio Test; RT, radiotherapy; CVD Events, post-RT cardiovascular events.

Figure S4. Kernel PCA of the Clinical Variable Set

Kernel PCA (RBF kernel) of the clinical variable set, with the K-means partition ($k = 2$); points indicate CDT status. The partition splits the cohort into clusters of 18 and 24; the lower-risk one contains 1 of the 10 events (NPV 94.4%, 17/18; 95% exact binomial CI: 72.7%–99.9%; specificity 53.1%) against a cohort-wide non-event rate of 76.2%.

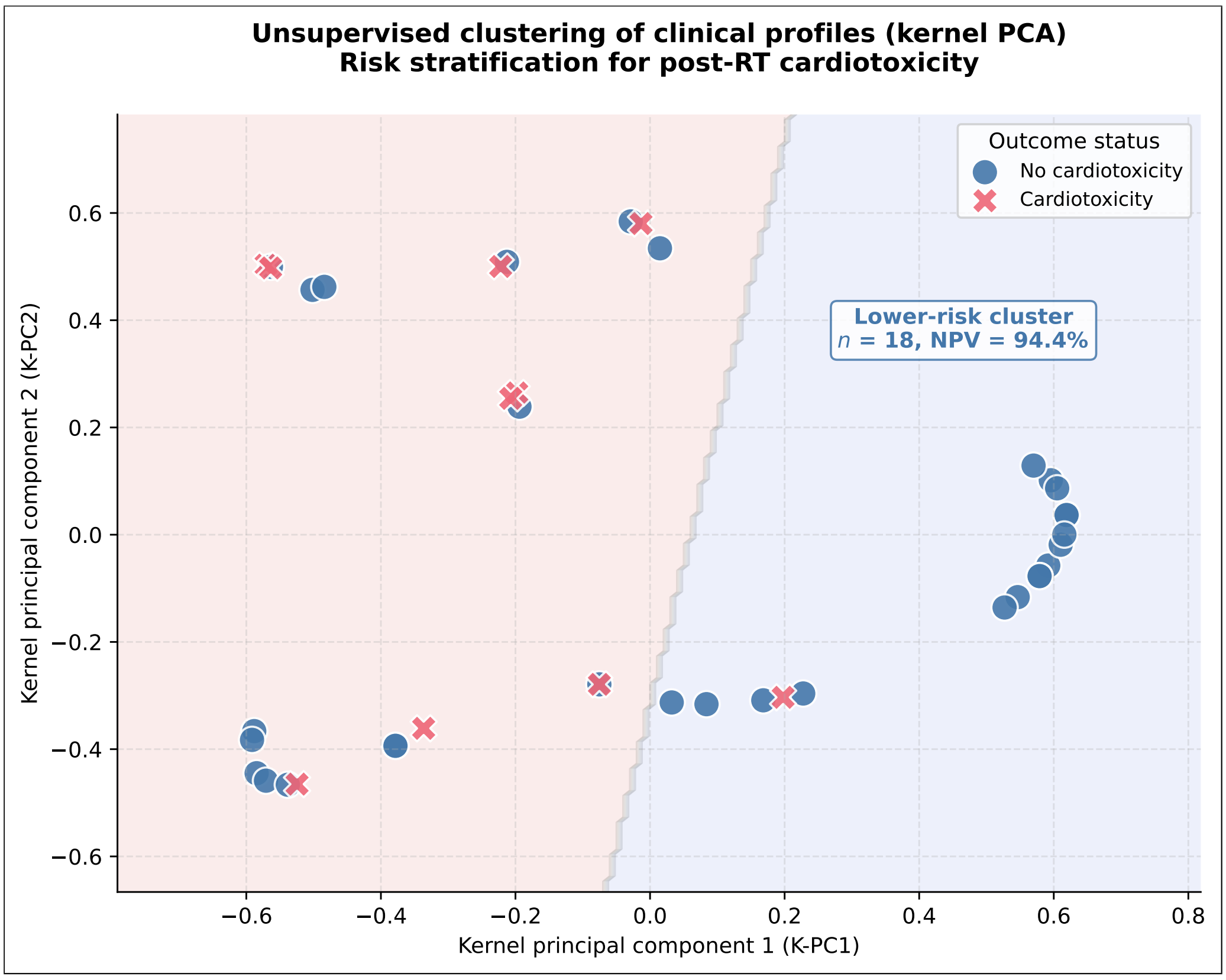

Figure S4. Kernel PCA (RBF kernel) of the clinical variable set, with the K-means partition ($k = 2$); points indicate CDT status. The partition splits the cohort into clusters of 18 and 24; the lower-risk one contains 1 of the 10 events (NPV 94.4%, 17/18; 95% exact binomial CI: 72.7%–99.9%; specificity 53.1%) against a cohort-wide non-event rate of 76.2%.

Figure S5. Calibration Plots (Combined Model)

Calibration plots for the Combined Model predicting (a) post-RT CDT and (b) post-RT CVD Events. Dashed line: perfect calibration; points: observed vs. predicted probability by risk quintile. Tick marks: individual predicted probabilities (green: events; grey: non-events).

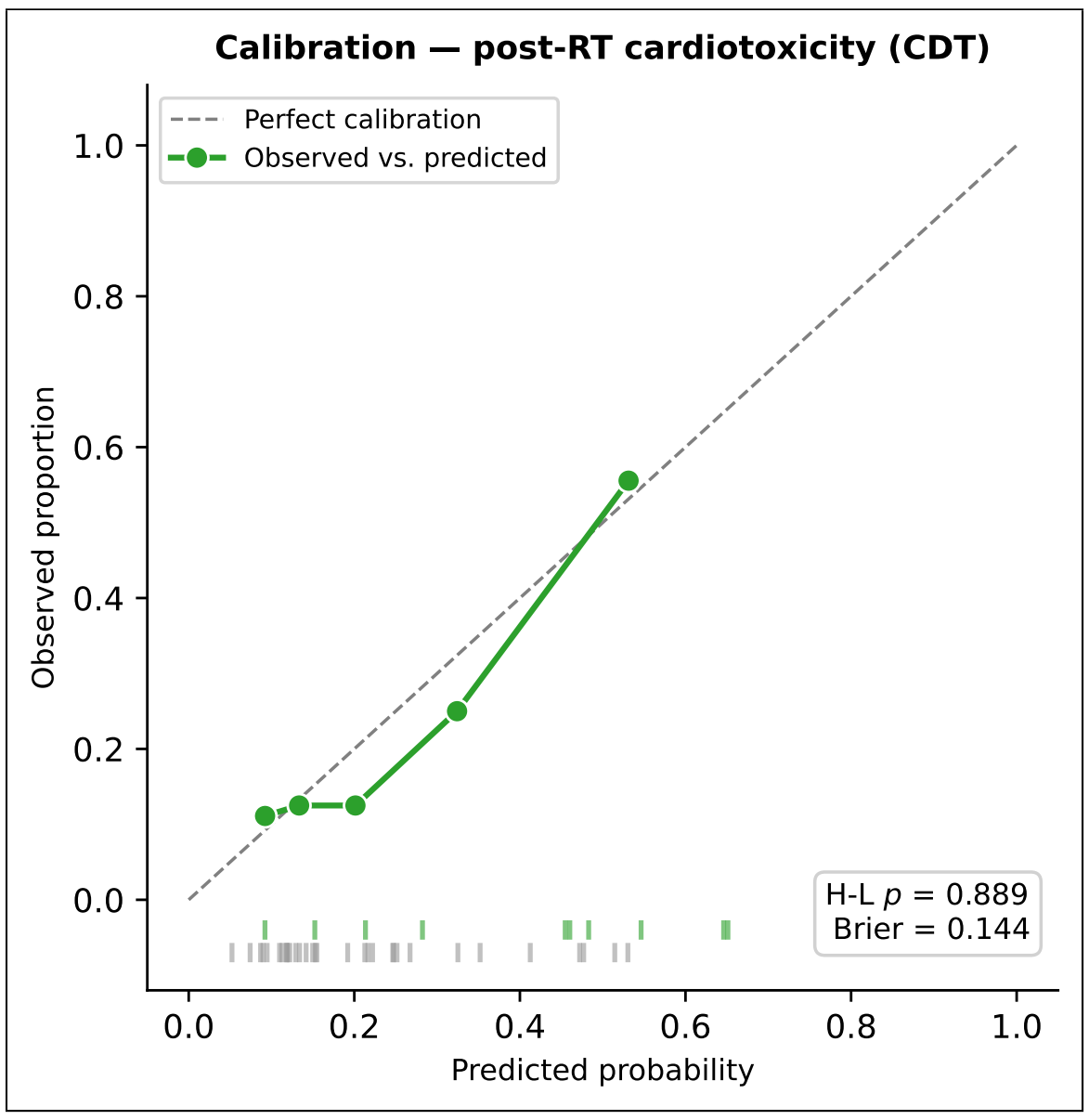

(a)

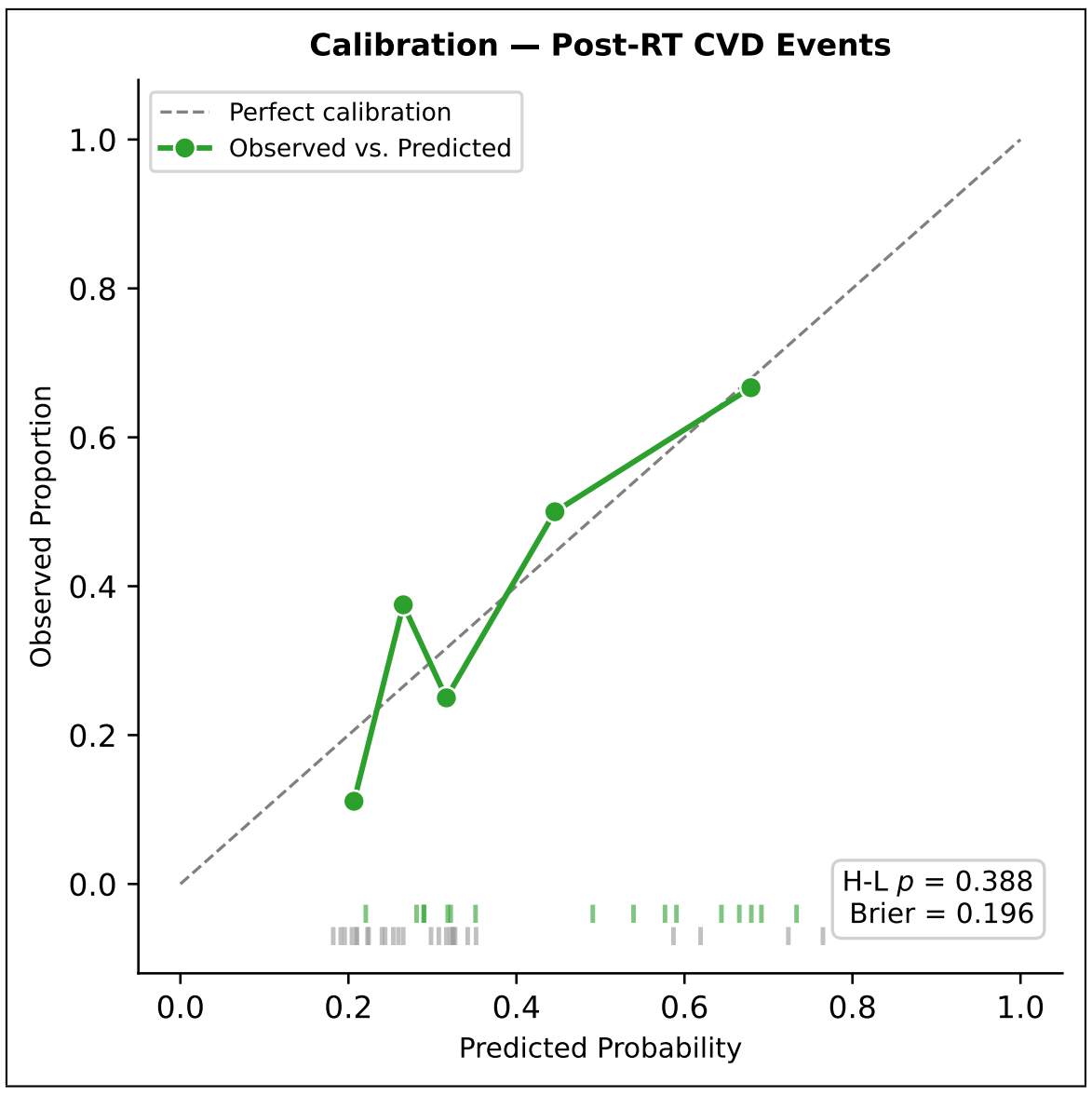

(b)

Figure S5. Calibration plots for the Combined Model predicting (a) post-RT CDT and (b) post-RT CVD Events. Dashed line: perfect calibration; points: observed vs. predicted probability by risk quintile. Tick marks: individual predicted probabilities (green: events; grey: non-events).

Figure S6. Kaplan–Meier CDT-free Survival by AHA PREVENT Category

Kaplan–Meier CDT-free survival stratified by AHA PREVENT category. No significant separation between categories (global three-category log-rank $p = 0.363$).

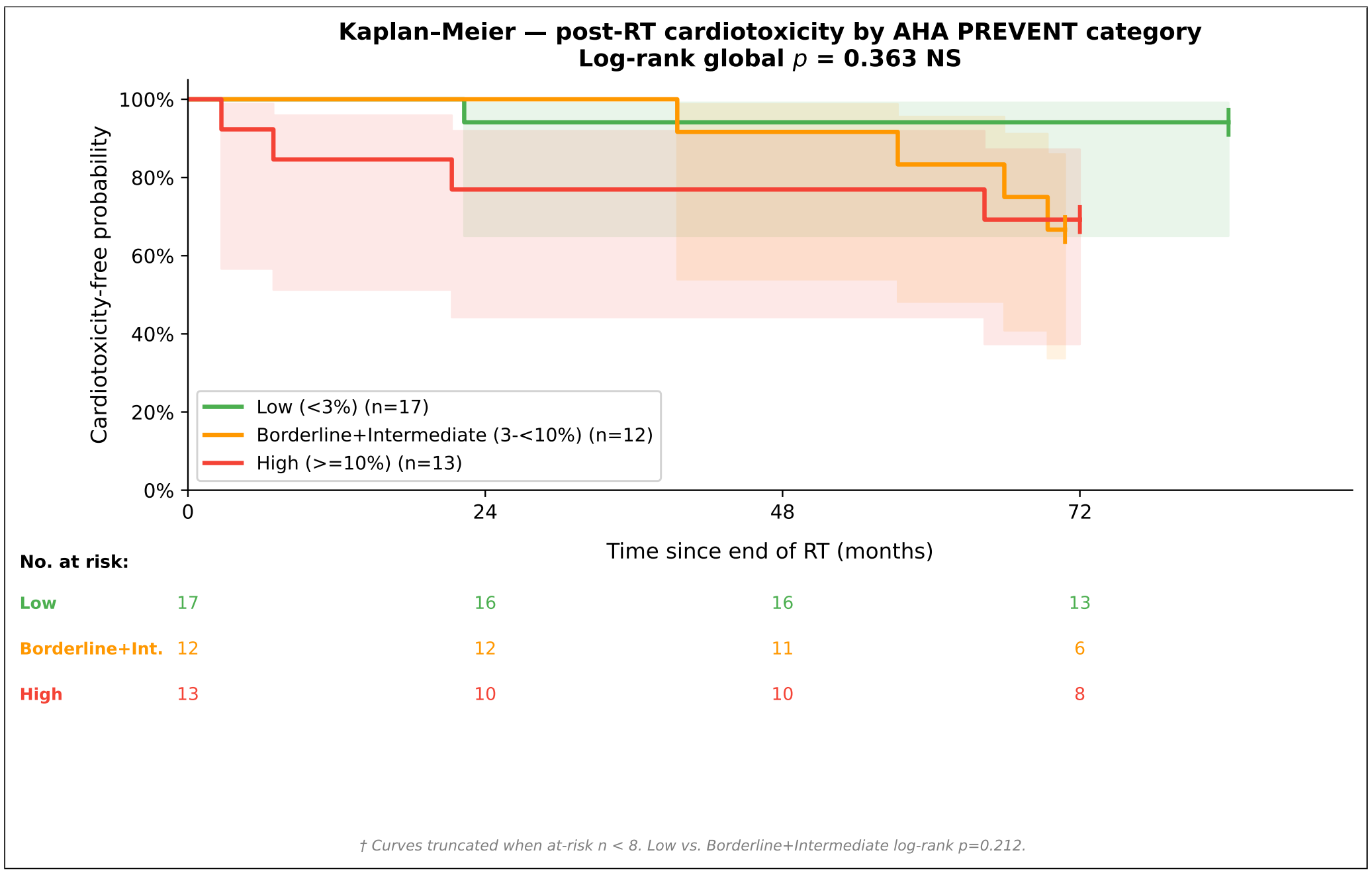

Figure S6. Kaplan–Meier CDT-free survival stratified by AHA PREVENT category, using time to the first CTCAE-graded event. No significant separation between categories (Low vs. Borderline+Intermediate log-rank $p = 0.212$; global three-category log-rank $p = 0.363$).

Table S11. Variance Explained by Principal Component

Table S11. Proportion of total variance explained by the first two principal components of each variable set ($n = 42$).

Variable Set	PC1	PC2	Cumulative
Set 1 — Clinical	42.7%	27.2%	69.9%
Set 2 — Dosimetric	79.9%	18.6%	98.5%

The near-complete variance concentration in Set 2 reflects the high collinearity between dose-volume metrics derived from the same organ.

Table S12. Prescribed Dose and Fractionation Schedules

Table S12. Prescribed dose, fractionation and equivalent dose in 2-Gy fractions (EQD2, $\alpha/\beta = 3$ Gy) for the cohort. Dose was recorded for 41 of 42 patients.

**Dose (Gy)**	**Fractions**	**Gy/fraction**	**EQD2 (Gy)**	**Patients**
40.0	15	2.67	45.3	25
42.5	16	2.66	48.1	8
50.0	25	2.00	50.0	5
42.4	16	2.65	47.9	1
30.0	5	6.00	54.0	1
48.0	40[a]	1.20	40.3	1
70.0	35	2.00	70.0	1

[a] Twice-daily hyperfractionation delivered as re-irradiation.
A boost to the tumour bed, when indicated, was delivered separately and is not included in the prescribed dose shown here.

Table S13. Sensitivity Analysis: Combined Model with LAD D_{mean}

Table S13. Firth penalized logistic regression for the combined model, substituting LAD D_{mean} for Heart V_5. Clinical variables unchanged.

	**Post-RT CDT**			**Post-RT CVD Events**		
**Variable**	**OR**	**95% CI**	p	**OR**	**95% CI**	p
Pre-RT Cardiac Disease	2.02	1.02–4.01	0.045*	1.84	0.94–3.59	0.076†
Hypertension	1.25	0.60–2.60	0.550	—	—	—
Age at RT	—	—	—	1.49	0.73–3.05	0.278
Heart D_{mean}	1.05	0.42–2.61	0.919	1.57	0.68–3.65	0.290
LAD D_{mean}	1.00	0.40–2.46	0.993	0.64	0.27–1.55	0.327
**AUC**	0.741 (vs. 0.766 primary)			0.796 (vs. 0.743 primary)		
**LRT vs. clinical**	$p = 0.19$			$p = 0.07$		

†$p < 0.10$; $*p < 0.05$. Dashes indicate variables not retained in that model. Models including LAD D_{mean} were fitted on the 41 patients with complete dosimetric data. Substituting LAD D_{mean} does not improve discrimination for CDT and LAD D_{mean} itself contributes no signal in either outcome, and pre-existing cardiac disease remains the only variable approaching conventional significance. Confidence intervals for LAD D_{mean} are considerably narrower than those for Heart V_5 in the primary model, reflecting the strong collinearity between Heart D_{mean} and Heart V_5.
